# TP8, A Novel Chondroinductive Peptide, Significantly Promoted Neo‐Cartilage Repair without Activating Bone Formation

**DOI:** 10.1002/adhm.202401752

**Published:** 2024-12-17

**Authors:** Mingjing Zhu, Siqing Jiang, Xingyang Li, Wenchao Zhong, Wei Cao, Qianting Luo, Antong Wu, Gang Wu, Qingbin Zhang

**Affiliations:** ^1^ Department of Temporomandibular Joint School and Hospital of Stomatology Guangdong Engineering Research Center of Oral Restoration and Reconstruction & Guangzhou Key Laboratory of Basic and Applied Research of Oral Regenerative Medicine Guangzhou Medical University Guangzhou 510180 China; ^2^ Department of Oral Cell Biology Academic Centre for Dentistry Amsterdam (ACTA) University of Amsterdam and Vrije Universiteit Amsterdam Amsterdam Movement Sciences Amsterdam 1081 LA the Netherlands; ^3^ Department of Human Genetics Amsterdam UMC Location Vrije Universiteit Amsterdam Amsterdam 1081 HZ Netherlands; ^4^ Department of Clinical Chemistry Amsterdam UMC Location Vrije Universiteit Amsterdam Amsterdam 1081 HV Netherlands; ^5^ Amsterdam Movement Sciences Tissue Function and Regeneration Amsterdam 1081 HV Netherlands; ^6^ Department of maxillofacial surgery Jiangmen Central Hospital Jiangmen 529030 China; ^7^ Savid School of Stomatology Hangzhou Medical College Hangzhou 311399 China

**Keywords:** cartilage tissue engineering, chondroinductive, peptides, TGF‐β3, TP8

## Abstract

The repair of large cartilage defects remains highly challenging in the fields of orthopedics and oral and maxillofacial surgery. A chondroinductive agent is promising to activate endogenous mesenchymal stem cells (MSCs) so as to facilitate cartilage regeneration. In this study, we analyze the crystallographic data of the critical binding domain of transforming growth factor β3 (TGF‐β3) with its type II receptor and successfully develop a novel chondroinductive peptide — TGF‐β3‐derived peptide No. 8 (TP8) that can induce an ectopic cartilage formation without obvious bone formation. TP8 shows a comparable capacity as TGF‐β3 in enhancing glycosaminoglycans (GAGs) and proteoglycans (PGs) secretion in the micromass of bone marrow MSCs (BMSCs) and promoting the expression of chondrogenic markers in comparison with the Control group. TP8 induces a significantly higher expression of the *SRY‐box transcription factor 9 (Sox9)* gene than TGF‐β3. Moreover, TP8 significantly upregulates the phosphorylation of Smad1/5 but not MAPK/JNK or Smad 2/3. The knockdown of low‐density lipoprotein receptor (LDLR) ‐related protein‐1 (Lrp1), a transmembrane endocytosis receptor, nullifies the TP8‐induced Sox9 expression. In the critical‐size cartilage defects in rabbit medial femoral condyles, TP8 can induce neo‐cartilage formation with a significantly thicker deep zone in comparison with the TGF‐β3 and Control. These findings suggest a promising application potential of TP8 in cartilage tissue engineering.

## Graphical abstract

This is the first study to successfully develop a novel peptide TP8, derived from TGF‐β3, and provide robust evidences to prove its chondroinductivity without osteoinductivity. TP8 enhances GAG and PG secretion, significantly upregulates *Sox9* gene expression, and promotes Smad1/5 phosphorylation. In rabbit cartilage defect models, TP8 induces neo‐cartilage formation with a significantly thicker deep zone compared to TGF‐β3 and Control.

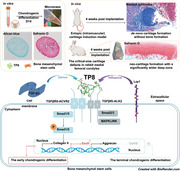

## 1 Introduction

Articular cartilage is a kind of aneural, avascular, highly hydrated, and chondrocyte‐containing connective tissue.^[^ ^1^ ^]^ It covers the epiphyseal surfaces of articulating bones and functions to lubricate joint surfaces and transfer mechanical loads to underlying subchondral bone.^[^ ^2^ ^]^ Articular cartilage bears a characteristic zonal structure, that is, superficial, middle, deep, and calcified zones.^[^ ^1^ ^]^ Articular cartilage can be damaged by a series of local and systemic diseases, such as osteoarthritis (OA), degeneration, and systemic immune diseases.^[^ ^3^ ^]^ Articular cartilage injuries are found in 60% to 66% of knees that undergo arthroscopy. The prevalence of focal full‐thickness chondral defects in these cases ranges from 4.2% to 6.2% and may be up to 36% in athletes.^[^ ^4^ ^]^ From 1990 to 2021, more than 500 million people worldwide have been affected by OA, with the prevalence of OA expected to rise from 26.6% to 29.5% by 2032.^[^ ^5^ ^]^ Due to the low density of reserve chondrocytes and limited cytokines surrounding defects, articular cartilage has very limited self‐regenerative potential, making the repair of large defects very challenging.^[^ ^6^ ^]^

To repair cartilage defects in the clinic, auto‐transplantation techniques such as mosaicplasty^[^ ^1^, ^7^ ^]^ and autologous chondrocyte implantation^[^ ^8^ ^]^ have been used to provide mature chondrocytes and cartilage matrix so as to repair cartilage defects. However, the application of these techniques is associated with limited available tissue, donor site pain and morbidity, graft failure, and stratification.^[^ ^1^, ^9^ ^]^ In fact, the articular joint has been recently shown to contain abundant mesenchymal stem cells (MSCs) in the surrounding tissues, such as cartilage, subchondral bone, adipose tissue, synovial fluid, and synovium even in the late stage of OA.^[^ ^10^ ^]^ This finding triggered the development of endogenous MSCs‐targeted cartilage regeneration and tissue engineering strategy, for which chondroinductive agents are of paramount importance to induce their proliferation and chondrogenic differentiation.^[^ ^11^ ^]^

Bone morphogenetic protein‐2 (BMP‐2) and transforming growth factor β3 (TGF‐β3) are two bioactive growth factors in the superfamily of transforming growth factor β (TGF‐β) and have long been shown to potently induce chondrogenic differentiation of MSCs.^[^ ^1^, ^12^ ^]^ BMP‐2 is crucial for the proliferation and maturation of chondrocytes during cartilage development in the growth plate.^[^ ^13^ ^]^ As an exogenous growth factor, BMP‐2 can initiate the chondrogenic lineage development of adult human MSCs in vitro^[^ ^14^ ^]^ and induce the formation of the characteristic zonal structure of cartilage in vivo.^[^ ^15^ ^]^ On the other hand, BMP‐2 is also a well‐established osteoinductive growth factor^[^ ^16^ ^]^ and human recombinant BMP‐2 has been already applied clinically for spine fusion and open tibial fracture healing.^[^ ^17^ ^]^ Due to its potent effect on promoting cartilage hypertrophy, calcification, and finally bone formation, exogenous BMP‐2 may not be valuable to be used as a therapeutic agent for cartilage formation or for cartilage restoration in OA.^[^ ^15^, ^18^ ^]^ TGF‐β3 contributes to cartilage physiology and pathology in a wide variety of aspects,^[^ ^19^ ^]^ as it can recruit MSCs,^[^ ^19^ ^]^ promote in vitro chondrogenesis,^[^ ^20^ ^]^ and induce in vivo neocartilage regeneration with zonal structure.^[^ ^21^ ^]^ However, TGF‐β3 has also been shown to predispose MSCs to hypertrophy.^[^ ^19^ ^]^ Furthermore, the usage of proteineous recombinant growth factors has several limitations, including expensive cost, poor production yield, and potential immunogenicity.^[^ ^22^ ^]^ In comparison, peptides can be chemically manufactured, which is associated with a much higher yielding rate, lower cost, and lower immunogenic risk.^[^ ^12b^ ^]^ An ideal chondroinductive peptide should not induce mineralization/calcification of cartilage or bone formation but be able to potently induce the chondrogenic differentiation of MSCs in vitro and the de novo cartilage formation with the characteristic zonal structure in vivo. However, hitherto, to our best knowledge, there is still a lack of experimentally‐proven chondroinductive peptides.^[^ ^23^ ^]^

In this study, we hypothesized that by segmenting the specific binding domain of TGF‐β3 with its receptors, we could design a real chondroinductive peptide. We designed and synthesized 10 unique TGF‐β3‐derived peptides (TPs) through analyzing the crystallographic data of the key binding domain of TGF‐β3 with its type II receptor.^[^ ^24^ ^]^ We screened chondroinductive and osteoinductive efficacy of the 10 TPs in an in vivo ectopic (intramuscular) bone/cartilage induction model in rats. We successfully found that TP No. 8 (TP8) induced a tremendous amount of de novo cartilage with zonal structure without obvious new bone formation. We further characterized the chondroinductive effect of the TP8 peptide using the micromass chondrogenesis model of mouse BMSCs in vitro and a critical‐size cartilage defect model in rabbit medial femoral condyles in vivo. Furthermore, we explored the underlying molecular mechanisms for its chondroinductivity.

## 2 Results

### 2.1 TP8 Induced de novo Cartilage Formation in the Ectopic Cartilage Model in Erector Spinae of SD Rats

**Figure 1:**
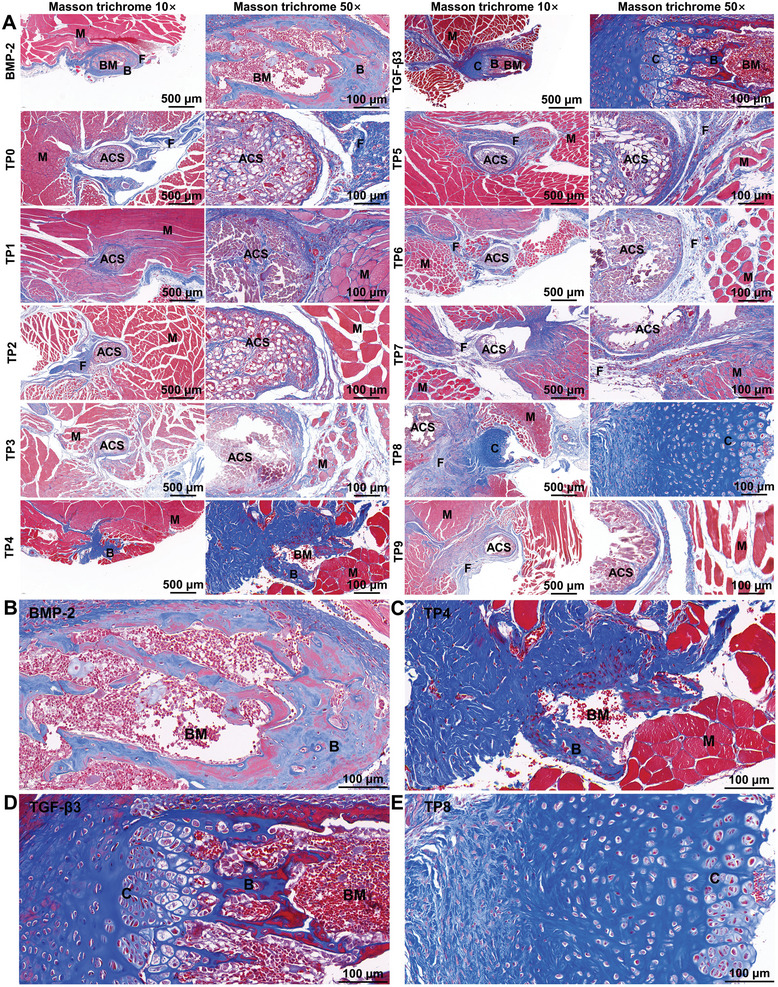
A) Light micrographs of Masson trichrome stained cross‐sections of the TGF‐β3, BMP‐2, and 10 TGF‐β3‐derived peptides (TPs) ‐containing collagen membrane that was implanted in erector spinae of Sprague–Dawley (SD) rats for 21 days. B–E) The enlarged light micrographs of Masson trichrome stained cross‐sections of BMP‐2, TP4, TGF‐β3, and TP8 groups containing collagen membrane. (M: muscle; B: bone; BM: bone marrow; C: chondroid matrix (cartilage); ACS: absorbable collagen sponge; F: fibrous tissue). Scale bar in *A* = 500 µm for 10× and 100 µm for 50×; scale bar in B–E = 100 µm.

The chondroinductivity of the 10 TPs, TGF‐β3, and BMP‐2 were investigated in an ectopic cartilage induction model in erector spinae of SD rats. Four weeks post‐implantation, the samples were retrieved and subjected to Micro‐CT analysis, which showed mineralized tissue formation in the groups of TGF‐β3, BMP‐2, and TP4. In contrast, no detectable mineralized tissue was detected in the other TP groups (Figure S1, Supporting Information). We performed histological analysis with Masson trichrome staining in the groups of 10 TPs, TGF‐β3, and BMP‐2 (**Figure** **1**A). Newly formed bone tissues containing osteoid, woven bone, active osteoblasts, and blood vessels were found in the groups of BMP‐2 and TP4 without detectable cartilage tissue (Figure 1B,C). In comparison, a well‐orchestrated osteochondral structure, that contained the interface between hypertrophying cartilage and immature woven bone was found in the TGF‐β3 group (Figure 1D). Strikingly, in the TP8 group, we found de novo cartilage tissue that contained a zonal structure and a thick deep zone without obvious bone tissue (Figure 1E; Figure S2, Supporting Information). The cartilage tissue was composed of a large number of mature chondrocytes. In contrast, no bone or cartilage tissue was found in the other TP groups.

### 2.2 TP8 Bore a Comparable Capacity in Inducing Chondrogenic Differentiation in the Micromasses of BMSCs as TGF‐β3

**Figure 2:**
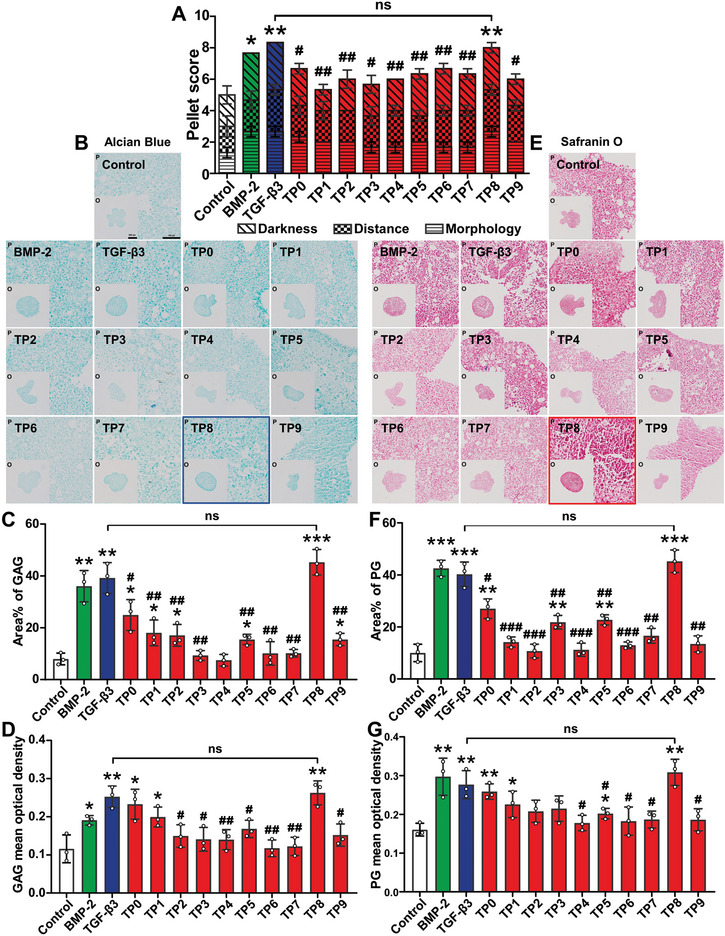
Light micrographs of Alcian Blue (B) and Safranin O (E) stained cross‐sections of the TGF‐β3, BMP‐2, and 10 TGF‐β3‐derived peptides (TPs) that were cultured in the micromass of mouse bone marrow stem cells (BMSCs) for 14 days. A) The pellet score in the TP8 group was comparable as that in the TGF‐β3 group, which was significantly higher than those in the groups of Control and the other TPs. C,D) The area percentage and optical density of glycosaminoglycans (GAGs) were confirmed quantitatively using Alcian blue staining. F,G) The area percentage and optical density of proteoglycans (PGs) were confirmed quantitatively using Safranin O staining. Scale bar in o of B and E = 500 µm; scale bar in *p* of B and E = 100 µm. (Data were presented as mean ± S.D.; statistical significance was calculated using one‐way ANOVA with a Tukey post‐test; significant effect of the treatment: *p < 0.05 versus Control group, **p < 0.01 versus Control group, ***p < 0.001 versus Control group, ^#^ *p* < 0.05 versus TGF‐β3, ^##^ *p* < 0.01 versus TGF‐β3, ^###^ *p *< 0.001 versus TGF‐β, *n* = 3/group).

We adopted the micromass model of BMSCs to assess the capacity of BMP‐2, TGF‐β3, and all the TPs in inducing in vitro chondrogenic differentiation. After a 14‐day treatment, complete, smooth, and resilient micromasses with round or ovary forms could be detected only in the presence of BMP‐2, TGF‐β3, and TP8. The micromasses in the groups of the other TPs showed irregular morphologies (**Figure** **2**B,E). The pellet score in the 500 ng mL^−1^ TP8 group was comparable as that in the 10 ng mL^−1^ TGF‐β3 group, which exhibited a considerably higher score than those in the groups of Control and the other TPs (Figure 2A). Consistently, the area percentage of glycosaminoglycans (GAGs) or proteoglycans (PGs) in the 500 ng mL^−1^ TP8 group was comparable as those in the 10 ng mL^−1^ TGF‐β3 group, which were much higher than those in the groups of Control and the other TPs (Figure 2C,F). Similar trends were also found in the optical densities of GAGs or PGs (Figure 2D,G).

### 2.3 Effect of TP8 on the Proliferation and Chondrogenic Differentiation of BMSCs

**Figure 3:**
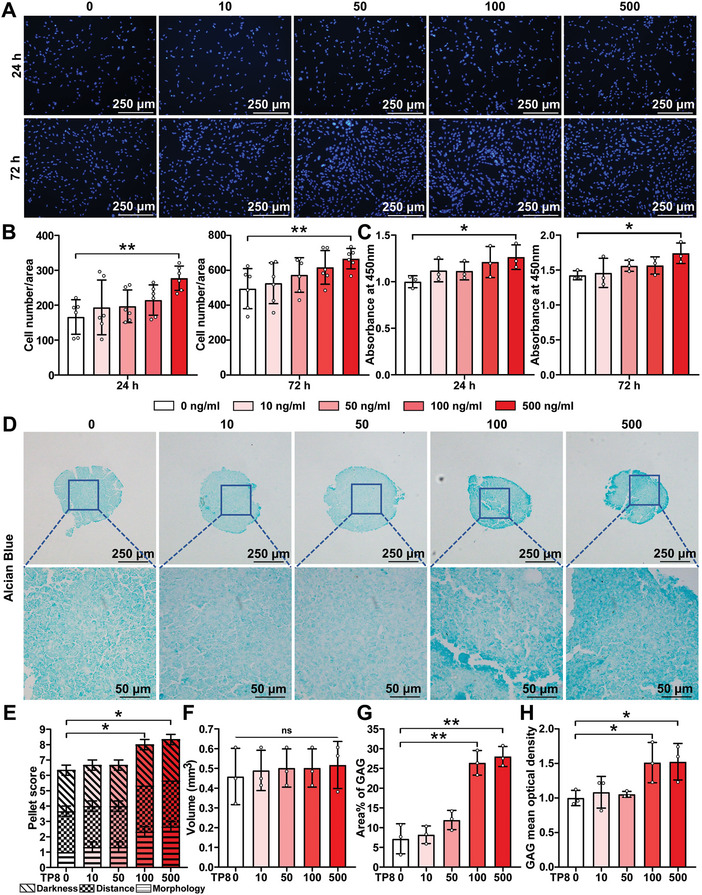
A) Fluorescent micrographs of Hoechst stained cross‐sections of TP8 within the concentration of 0 to 500 ng mL^−1^ that were cultured in the micromass of mouse bone marrow stem cells (BMSCs) for 24 and 72 h. B) Figure showing that 500 ng mL^−1^ TP8 significantly enhanced the number of BMSCs in comparison with the Control group (0 ng mL^−1^) at 24 and 72 h. C) Figure showing that the effects of TP8 on cell proliferation and viability appeared a dose‐dependent increasing trend within the concentration of 0 to 500 ng mL^−1^ at 24 and 72 h in culture using CCK8 assay. TP8 (500 ng mL^−1^) enhanced the number of BMSCs in comparison with the Control group (0 ng mL^−1^) at 24 and 72 h. D) Light micrographs of Alcian Blue stained cross‐sections of the TP8 within the concentration of 0 to 500 ng mL^−1^ that were cultured in the micromass of mouse bone marrow stem cells (BMSCs) for 14 days. E) The pellet score of micromass showing that in the groups of 100 and 500 ng mL^−1^ were significantly higher than that in the Control group (0 ng mL^−1^). F) No significant difference in the size of micromass was found among the different doses of TP8. 100 or 500 ng mL^−1^ TP8 robustly enhanced the percentage area (G) and optical density (H) of GAGs in comparison with the Control group. Scale bar in *A* = 250 µm; scale bar in *D* = 250 and 50 µm. (Data were presented as mean ± S.D.; statistical significance was calculated using one‐way ANOVA with a Tukey post‐test; significant effect of the treatment: *p < 0.05, **p < 0.01, *n* = 3/group).

The dose‐dependent effect of TP8 upon the viability, proliferation, and chondrogenic differentiation of BMSCs was assessed by utilizing the Hoechst staining assay, CCK‐8 assay, pellet score, and Alcian Blue staining. Within the concentration of 0 to 500 ng mL^−1^, the effects of TP8 on cell viability and proliferation showed a dose‐dependent increasing trend. 500 ng mL^−1^ TP8 significantly enhanced the viability and proliferation of BMSCs in comparison with the Control group (0 ng mL^−1^) (**Figure** **3**A–C; Figure S3, Supporting Information). After a 14‐day treatment, as shown by Alcian blue staining (Figure 3D), the pellet scores in the groups of 100 and 500 ng mL^−1^ were significantly higher than those in the Control group (Figure 3E). There was no discernible difference in the size of the micromass among the different doses of TP8 (Figure 3F). TP8 (100 or 500 ng mL^−1^) robustly enhanced the area percentage and optical density of GAGs in comparison with the Control group. (Figure 3G,H). According to the above results, 500 ng mL^−1^ TP8 was chosen in the subsequent experiments.

**Figure 4:**
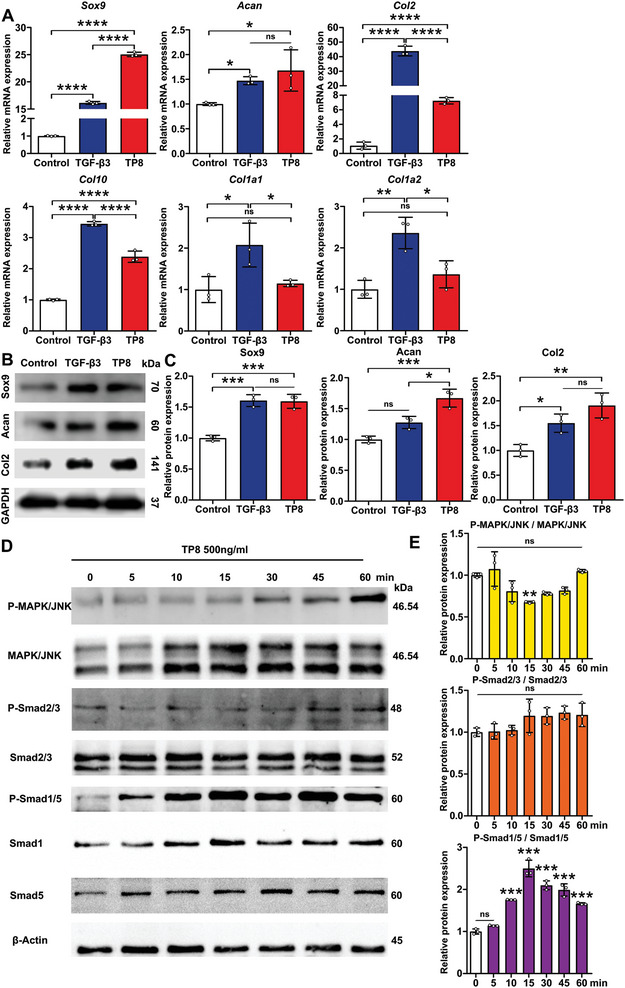
A) Figure showing the relative gene expression of *Sox9*, *Col2*, *Acan, Col10, Col1a1*, and *Col1a2* in BMSCs after a 14‐day treatment. B) Figure showing the protein expression of Sox9, Col2, and Acan in BMSCs after a 7‐day treatment. C) Quantification of western blot. D) Figure showing that TP8 remarkedly enhanced the level of p‐Smad1/5 but not p‐Smad2/3 or p‐MAPK/JNK within a 60‐min monitoring span. E) Quantification of western blot. (Data were presented as mean ± S.D.; statistical significance was calculated using one‐way ANOVA with a Tukey post‐test; significant effect of the treatment: *p < 0.05 versus, **p < 0.01, ***p < 0.001, ****p < 0.0001, *n* = 3/group).

Fourteen days post‐treatment, TP8 significantly increased the expression of all the selected chondrogenic genes, such as *Sox9*, *Col2*, and *Acan*. Interestingly, 500 ng mL^−1^ TP8 could induce a significantly higher expression of the *Sox9* gene (1.6‐folds), but a significantly lower expression of the *Col10* gene (0.7‐folds) than 10 ng mL^−1^ TGF‐β3. TGF‐β3 significantly increased the gene expression of *Col1a1* and *Col1a2* in comparison with the Control group, while TP8 didn't significantly change the expression of *Col1a1* gene and *Col1a2* genes (**Figure** **4**A). Western blot analysis showed that TP8 induced  higher expression of Sox9, Col2, and Acan proteins in comparison with Control 7 days post‐treatment (Figure 4B,C). Furthermore, TP8 remarkably enhanced the level of p‐Smad1/5 but not p‐Smad2/3 or p‐MAPK/JNK within a 60‐min monitoring span (Figure 4D,E).

### 2.4 Spatial Distribution and Potential Receptors of TP8 on BMSCs

**Figure 5:**
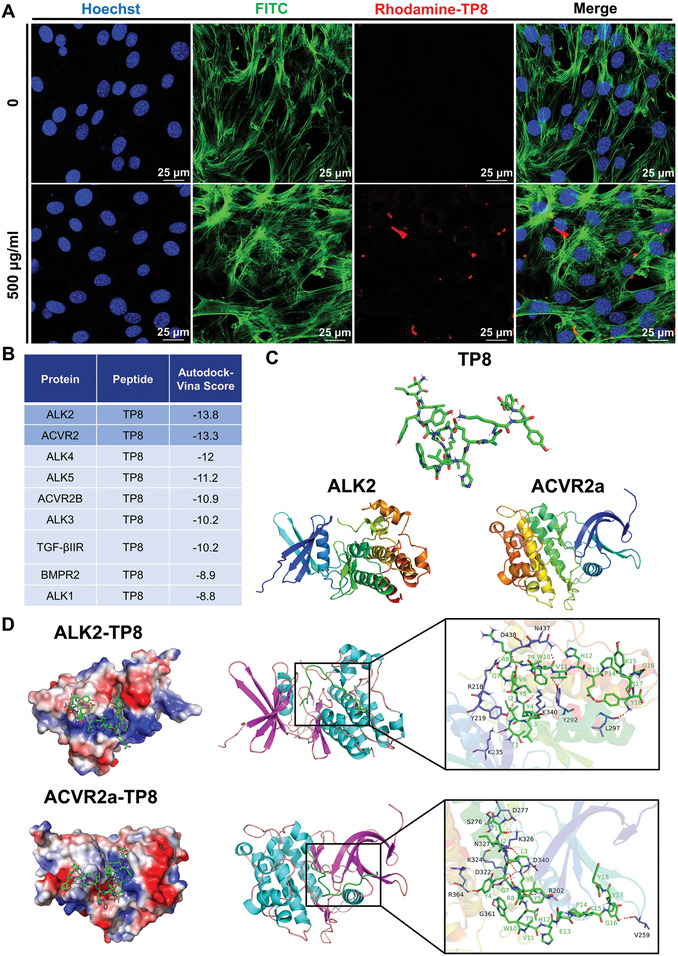
A) Fluorescent micrographs showing that after fixation and staining for nuclei (Hoechst, blue), actin filaments (FITC‐Phalloidin, green), and TP8 (Rhodamine, red), rare intracellular distribution of TP8 within BMSCs. B) Figure showing the docking scores of TP8 with those nine TGFβ receptors (ALK2, ACVR2, ALK4, ALK5, ACVR2B, ALK3, TGFβR2, BMPR2 and ALK1). C) Schematic illustrating the structure model of TP8, ALK2, and ACVR2a. D) Schematic illustrating the surface (left column) and 3D (middle column) binding model of TP8 with ALK2 and ACVR2a. In the left column, the TP8 was colored in green, red represents negatively charged surfaces, and blue represents positively charged surfaces. In the right column, the residues in TP8 and protein (ALK2 and ACVR2a) were depicted as green and blue sticks, respectively, and the red dashed lines represent hydrogen bond interactions. Scale bar in *A* = 25 µm.

Confocal laser scanning microscope (CLSM) images showed rare intracellular distribution of TP8 within BMSCs (**Figure** **5**A). Therefore, TP8 might take effect by interacting with transmembraneous receptors. The molecular docking analysis revealed that TP8 exhibited docking scores below ‐5.0 with all nine TGFβ receptors (ALK2, ACVR2, ALK4, ALK5, ACVR2B, ALK3, TGFβR2, BMPR2, and ALK1), indicating a notable binding affinity with each of these receptors. The binding energy of ALK2 and ADVR2 with TP8 was the most negative, which suggested that TP8 might preferably bind with these two receptors (Figure 5B–D).

### 2.5 mRNA Sequencing Analysis

**Figure 6:**
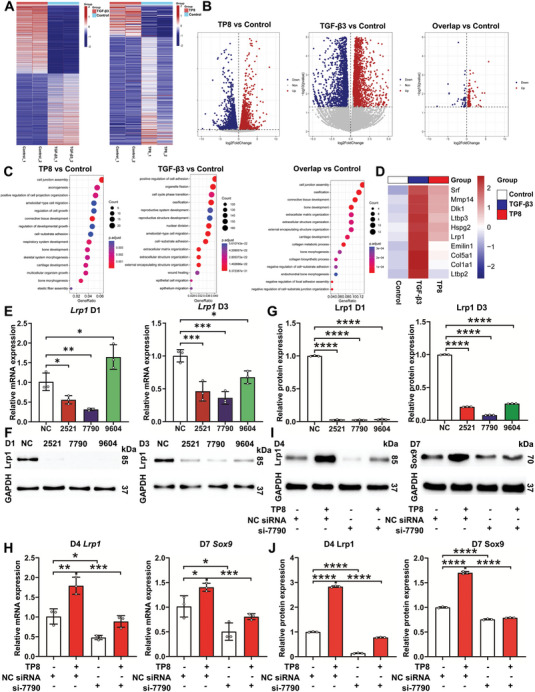
A) Heatmap showing the clustering performed for differentially expressed genes between TP8, TGF‐β3, and Control group. B) Volcano map showing the differentially expressed genes in TP8‐treated mouse BMSCs, TGF‐β3‐treated mouse BMSCs, and overlap of TP8‐treated mouse BMSCs and TGF‐β3‐treated mouse BMSCs group in comparison with the BMSCs in the Control group. C) Figure showing the “GO‐Process” enrichment analyzed that the differentially expressed signaling pathways in TP8‐treated mouse BMSCs, TGF‐β3‐treated mouse BMSCs, and overlap of TP8‐treated mouse BMSCs and TGF‐β3‐treated mouse BMSCs group in comparison with the BMSCs in the Control group. D) Heatmap showing the differentially expressed chondrogenesis‐related genes in the overlap of TP8‐treated mouse BMSCs and TGF‐β3‐treated mouse BMSCs group in comparison with the BMSCs in the Control group. Figure showing that the expression of gene *Lrp1* (E) and the protein Lrp1 (F) were significantly down‐regulated by all the selected siRNAs (si‐2521, si‐7790, and si‐9604) after 1 day and 3 days post‐treatment. G) Quantification of western blot. H) Figure showing that the presence of si‐7790 nullified the promoting effect of TP8 on the gene expressions of *Lrp1* and *Sox9*. I) Figure showing that si‐7790 also nullified the promoting effect of TP8 on the protein expressions of Lrp1 and Sox9. J) Quantification of western blot. (Data were presented as mean ± S.D.; statistical significance was calculated using one‐way ANOVA with a Tukey post‐test; significant effect of the treatment: *p < 0.05, **p < 0.01, ***p < 0.001, ****p < 0.0001, *n* = 3/group).

To explore the molecular mechanisms for the chondroinductive differentiation of TP8, we performed the sequencing of the whole transcriptome and analysis to assess the gene expressions among the BMSCs treated with or without TGF‐β3 or TP8 for 1 day. The results of RNA sequencing analysis indicated that in comparison with the BMSCs in the Control group (no TGF‐β3 or TP8), 2507 mRNAs were upregulated and 2377 mRNAs were downregulated in TGF‐β3‐treated mouse BMSCs. TP8‐treated mouse BMSCs were found to have 356 significantly upregulated and 104 downregulated mRNAs when compared to the BMSCs in the Control group. Furthermore, 111 mRNAs were upregulated and 44 mRNAs were downregulated in the overlap of TGF‐β3‐treated mouse BMSCs and TP8‐treated mouse BMSCs in comparison with the BMSCs in the Control group (**Figure** **6**A,B). A “GO‐Process” enrichment analysis was performed in order to determine the functional differences among the differential genes. TGF‐β3 and TP8 induced a series of common signaling pathways, such as cartilage development, cell junction assembly, regulation of cell growth, and connective tissue development (Figure 6C). Furthermore, we screened the chondrogenesis‐related genes that were robustly upregulated by both TP8 and TGF‐β3, among which Lrp1 attracted our attention since it is a transmembrane receptor (Figure 6D).

### 2.6 Effects of Lrp1 Knockdown on TP8‐Induced Chondrogenic Differentiation of BMSCs

To identify the effect of Lrp1 on TP8‐induced chondrogenic differentiation, we knocked down the Lrp1 using siRNA (si‐2521, si‐7790, and si‐9604, **Table** **1**) transfection in BMSCs. The expressions of the *Lrp1* gene and protein were significantly downregulated by all the selected siRNAs (Figure 6E–G). Since si‐7790 exhibited the best efficacy in down‐regulating the expression of gene *Lrp1* and the protein Lrp1 in BMSCs, it was used in further experiments. The presence of si‐7790 nullified the promoting effect of TP8 on the gene expressions of *Lrp1* and *Sox9*. Consistently, si‐7790 also nullified the promoting effect of TP8 on the protein expressions of Lrp1 and Sox9. The gene expression of *Lrp1* was significantly down‐regulated by si‐7790 in BMSCs of both NC siRNA group (0.48‐folds) and TP8 group (0.49‐folds) on day 4 (Figure 6H). The expression of Lrp1 protein was also dramatically downregulated after Lrp1 knockdown in comparison with the BMSCs in both the NC siRNA group (0.14‐folds) and TP8 group (0.27‐folds) on day 4 (Figure 6I,J). Under the same conditions, the knockdown of Lrp1 nullified the promoting effect of TP8 on the expression of the *Sox9* gene and Sox9 protein on day 7 (Figure 6H–J).

**Table 1:** siRNA sequences.

Name	Sequence (5′‐3′)
Lrp1‐2521	GCUCACACCGAGAUAUCUUTT
Lrp1‐7790	GCAGCGAGCCAACAAGUAUTT
Lrp‐9604	CCAACUACACACUGCUUAATT
NC	UUCUCCGAACGUGUCACGUTT

### 2.7 TP8‐Functionalized GelMA Hydrogels Maintain the Viability of the Encapsulated BMSCs

**Figure 7:**
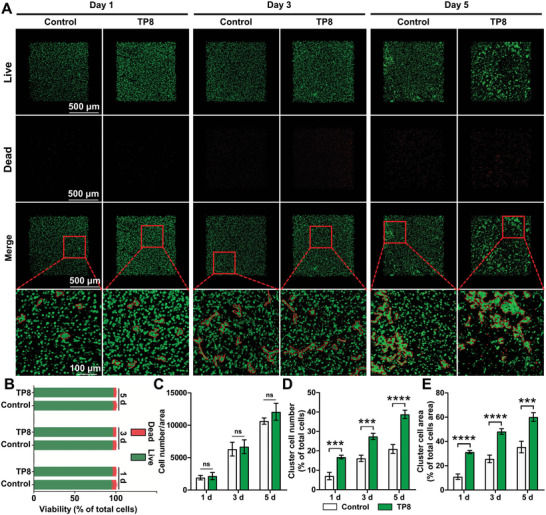
A) Fluorescent micrographs showing that after fixation and live/dead staining for 1, 3, and 5 days, BMSCs grew evenly in the TP8‐functionalized GelMA hydrogels and a few cells were stained fluorescent red (dead cells). B) The quantification analysis of the percentage of the number of live and dead cells in the total number of cells. C) The quantification analysis of the number of live cells. D) The quantification analysis of the number of cluster cells. E) The quantification analysis of the area of cluster cells. (Data were presented as mean ± S.D.; statistical significance was calculated using unpaired *t‐*test; significant effect of the treatment: ***p < 0.001 versus Control group, ****p < 0.0001 versus Control group, *n* = 3/group). Scale bar in *A* = 500 and 100 µm.

We further evaluated the effect of TP8 on the chondrogenic differentiation of BMSCs in a 3D culture system − GelMA hydrogel using live/dead fluorescence staining. The staining results of live/dead BMSCs in the TP8‐functionalized GelMA hydrogels are shown in **Figure** **7**A (fluorescence staining of live/dead cells), Figure 7B (proportion of living and dead cells to total cells), Figure 7C (the number of live cells), and Figure 7D,E (the number and area of cluster cell), BMSCs grew evenly in the TP8‐functionalized GelMA hydrogels and only a few cells were stained fluorescent red (dead cells). The number of cells rose from day 1 to day 5 and tended to cluster with time. Each time point showed the percentages of living cells in all the groups reached 95%, demonstrating no significant effect of TP8 on cell viability. On each time point, the cluster cell number and area in the TP8 group were significantly higher than those in the corresponding Control group.

### 2.8 TP8 Promoted the Repair of Articular Cartilage Defects In Vivo

Cartilage defects with a diameter of 4.0 mm and a depth of 2.5 mm were successfully created on the trochlear groove of the knee. TP8‐functionalized GelMA hydrogels, with or without TP8, were implanted to repair these defects. All rabbits survived the surgical procedure and postoperative treatment, returning to normal activity without any infections at the surgical or intra‐articular implantation sites. Rabbits were sacrificed and the whole cartilage tissues were harvested for gross and histological evaluation 4 weeks post implantation.

**Figure 8:**
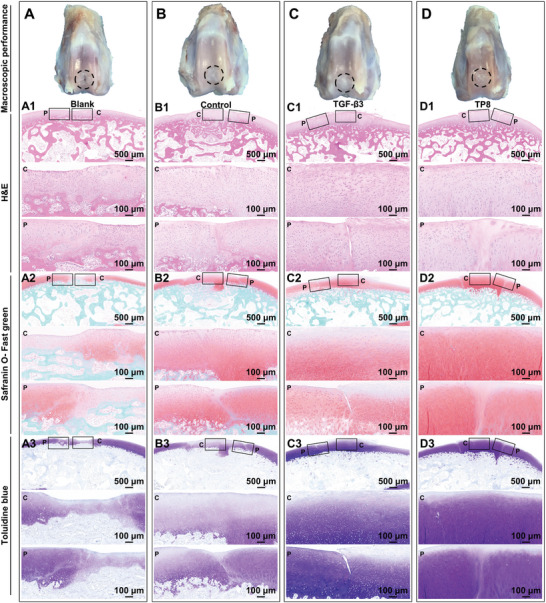
Macroscopic images and light micrographs of hematoxylin and eosin (H&E), Safranin‐O and Toluidine blue stained cross‐sections of the Blank (no implanted material) (A), Control group implanting with GelMA hydrogel without TP8 (B), TGF‐β3‐ functionalized GelMA hydrogel (C) and TP8‐ functionalized GelMA hydrogel (D) that were implanted in medial femoral condyle cartilage defect in rabbits for 4 weeks. (p, indicates the periphery of the defect; c, indicates the center of the defect) Scale bar in A1–D3 = 500 µm; scale bar in p and c = 100 µm.

**Figure 9:**
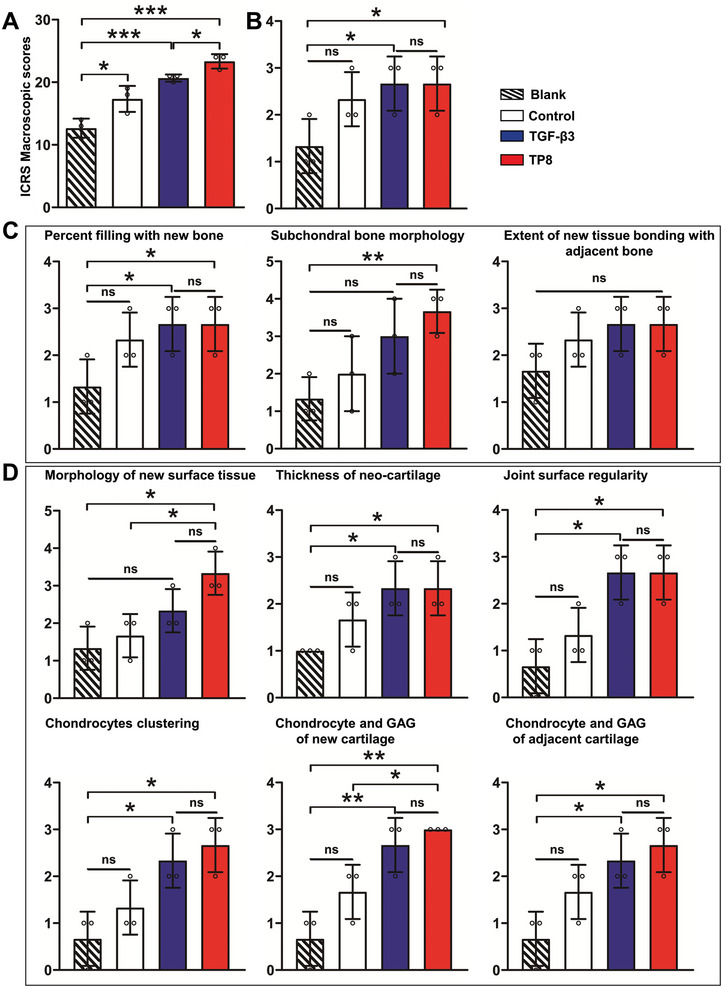
A) Figure showing the Macroscopical International Cartilage Repair Association (ICRS) scores of the repaired tissue. B) Figure showing the histological scores system of overall defect evaluation of the repaired tissue. C) Figure showing the histological scores system for subchondral bone evaluation of the repaired tissue. D) Figure showing the histological scores system for cartilage evaluation of the repaired tissue. (Data were presented as mean ± S.D.; statistical significance was calculated using one‐way ANOVA with a Tukey post‐test; significant effect of the treatment: *p < 0.05, **p < 0.01, ***p < 0.001, *n* = 3/group).

Macroscopically, 4 weeks post‐surgery, in the Blank group (no implanted material), the defects didn't heal completely with a distinct edge from the surrounding cartilage (**Figure** **8**A). In the control group (GelMA without TGF‐β3 or TP8), the defects were dominated by white fibrous cartilage‐like tissue. (Figure 8B). Conversely, the defects in the TGF‐β3 group (Figure 8C) and the TP8 group (Figure 8D) were mainly filled with hyaline cartilage, which bore a similar morphology and color to the adjacent cartilage tissue. Furthermore, the repairing quality was semi‐quantitatively evaluated using the International Cartilage Repair Society (ICRS) macroscopic evaluation of cartilage repair and histological scoring system for evaluation of the repair of full‐thickness articular cartilage defect in rabbits (Tables S1, S2, Supporting Information).^[^ ^25^ ^]^ The TP8 group had a significantly higher ICRS macroscopic score than the other three groups (**Figure** **9**A). For all the parameters in the histological scoring system, including the overall defect evaluation scores (Figure 9B), subchondral bone evaluation (filling and morphology) (Figure 9C), and cartilage evaluation (surface, thickness, joint surface regularity, chondrocytes clustering, chondrocytes, and GAG of adjacent cartilage) (Figure 9D), no significant difference was found between TP8 and TGF‐β3. Except for the parameter of the extent of new tissue bonding with adjacent bone, the TP8 group exhibited a significantly higher level of performance than the Blank group. In comparison, TGF‐β3 was not superior to the Blank group for the parameters of subchondral bone morphology, extent of new tissue bonding with adjacent bone, and morphology of new surface tissue. For all the parameters, no significant differences were found between the Control group and the Blank group.

**Figure 10:**
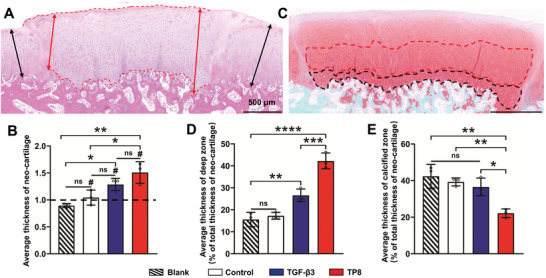
A) Light micrographs of H&E stained cross‐section of TP8 that was implanted in medial femoral condyle cartilage defect in rabbits for 4 weeks. Neo‐cartilage thickness evaluation by using average thickness of neo‐cartilage (red rows: thickest thickness and thinnest thickness) / average thickness of surrounding normal cartilage (black rows: two sides of surrounding normal cartilage). B) Figure showing that the cartilage thickness in the TP8 group but not in the TGF‐β3 group was significantly higher than that of the Control group. C) Light micrographs of Safranin‐O stained cross‐section of TP8 that was implanted in medial femoral condyle cartilage defect in rabbits for 4 weeks. Deep and calcified zone thickness evaluation by using the average thickness of deep and calcified zone thickness. The red curve showed the deep zone area and the black curve showed the calcified zone area. D) Figure showing that the deep zone accounts for ≈40% of articular cartilage thickness in the TP8 group, which was significantly higher than that (25%) in the TGF‐β3 group. E) Figure showing that the percentages of the calcified zones in the Control, Blank, and TGF‐β3 groups ranged from 36% to 43%, while the calcified zone in the TP8 group comprised only ≈20% of the total articular cartilage thickness. (Data were presented as mean ± S.D.; statistical significance was calculated using one‐way ANOVA with a Tukey post‐test; significant effect of the treatment: *p < 0.05, **p < 0.01, ***p < 0.001, ****p < 0.0001, *n* = 3/group).

Additionally, the cartilage thickness in the Blank group was less than that of surrounding cartilage tissue, and the Control group did not significantly enhance the cartilage thickness. In comparison, the cartilage thicknesses in the TGF‐β3 and TP8 groups were much higher than the average thickness of surrounding cartilage. Meanwhile, the cartilage thickness in the TP8 group but not in the TGF‐β3 group was significantly higher than that of the Control group (**Figure** **10**A,B). In the TGF‐β3 group, a large portion of chondrocytes showed spherical hypertrophic morphology, while the chondrocytes in the TP8 group exhibited more characteristic flat and longitudinally‐aligned morphology (Figure 10C). Histomorphometric analysis showed that in the TP8 group, the deep zone makes up ≈40% of the articular cartilage thickness, which is much more than that (25%) in the TGF‐β3 group (Figure 10D). In contrast, the percentages of deep zones in the Control and Blank group were only ≈15%. Furthermore, the percentages of the calcified zones in the Control, Blank, and TGF‐β3 groups ranged from 36% to 43%, while the calcified zone in the TP8 group comprised only ≈20% of the total articular cartilage thickness (Figure 10E).

**Figure 11:**
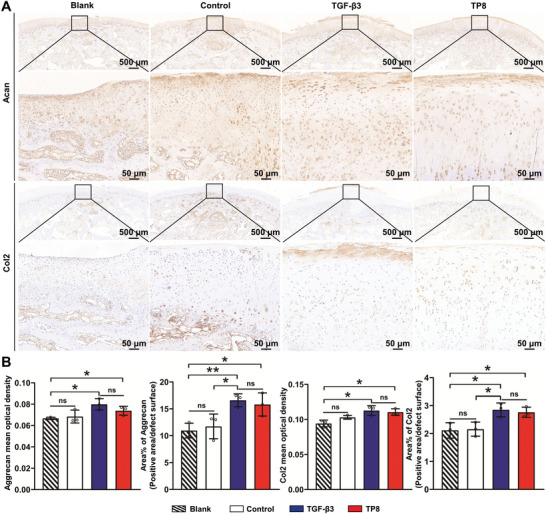
A) Light micrographs of immunohistochemically stained cross‐sections (Acan and Col2) of the Blank (no implanted material), Control group implanting with GelMA hydrogel without TP8, TGF‐β3‐ functionalized GelMA hydrogel and TP8‐ functionalized GelMA hydrogel that was implanted in medial femoral condyle cartilage defect in rabbits for 4 weeks. B) Figure showing that the optical densities and area percentage of Acan and Col2 in the TGF‐β3 and TP8 groups were significantly higher than those in the Blank and Control groups. No significant differences in these parameters were found between the TP8 group and the TGF‐β3 group. Scale bar in A = 500 and 50 µm. (Data were presented as mean ± S.D.; statistical significance was calculated using one‐way ANOVA with a Tukey post‐test; significant effect of the treatment: *p < 0.05, **p < 0.01, *n *= 3/group).

Immunohistochemical staining was conducted to detect Acan and Col2 to evaluate the effects of TP8 on in vivo chondrogenesis (**Figure** **11**A). Four weeks post‐surgery, the optical densities and area percentages of Acan and Col2 in the TGF‐β3 and TP8 groups were significantly higher than those in the Blank and Control groups. No significant differences in these parameters were found between the TP8 group and the TGF‐β3 group (Figure 11B).

## 3 Discussion

An ideal chondroinductive peptide should not induce mineralization/calcification of cartilage or bone formation but possess the capacity to potently induce the chondrogenic differentiation of MSCs in vitro and the de novo cartilage formation with the characteristic zonal structure of the cartilage in vivo. In this study, we successfully developed a real chondroinductive peptide — TP8 by showing that it could induce ectopic cartilage formation without obvious bone formation. TP8 (500 ng mL^−1^) showed a comparable capacity as 10 ng mL^−1^ TGF‐β3 in enhancing GAGs and PGs secretion in the micromass of BMSCs and promoting the expression of chondrogenic markers (Sox9, Acan, and Col2) in comparison with the Control group. In particular, 500 ng mL^−1^ TP8 induced a significantly higher expression of the *Sox9* gene than 10 ng mL^−1^ TGF‐β3. Moreover, TP8 significantly upregulated the phosphorylation of Smad1/5 but not MAPK/JNK or Smad 2/3. The knockdown of Lrp1 nullified the TP8‐induced Sox9 expression. In rabbit medial femoral condyles with critical‐size cartilage defects, TP8 could induce neo‐cartilage formation with a significantly thicker deep zone and thinner calcified zone in comparison with the TGF‐β3 and Control. These findings imply that TP8 has a promising potential in cartilage tissue engineering applications.

In the field of cartilage tissue engineering, various peptides have been developed from cartilage cell–cell adhesion molecule / extracellular matrix and growth factors. For example, the conserved three‐amino acid sequence HAV from N‐cadherin, a calcium‐dependent type‐1 transmembrane glycoprotein that promotes cell–cell adhesion junctions during tissue growth, migration, and differentiation, is incorporated into N‐cadherin mimetic peptide (Ac‐HAVDIGGGC). This peptide facilitates cell‐to‐cell adhesion by functioning as a homophilic region for cell adhesion recognition.^[^ ^26^ ^]^ Over a period of 28 days, hyaluronic acid hydrogels functionalized with the N‐cadherin mimetic peptide significantly increased the number of GAGs and total collagen content in encapsulated MSCs by 1.5 and 1.8 times, respectively, in comparison to hydrogels functionalized with scrambled N‐cadherin mimetic peptides (Ac‐AGVGDHIGC).^[^ ^27^ ^]^ Nevertheless, the promotion of chondrogenesis in vitro and in vivo by N‐cadherin mimetic peptide‐conjugated materials occurs exclusively with TGF‐β3 present, indicating that the N‐cadherin mimetic peptide alone lacks chondroinductive properties.^[^ ^28^ ^]^ Originally identified in fibronectin, RGD binds to integrins on the cell membrane to facilitate cell‐ECM adhesion, spreading, and various other cellular activities.^[^ ^29^ ^]^ As the minimal functional sequence, RGD is typically linked with different amino acid sequences to form RGD peptides, such as GGGGRGDY, GCGYGRGDSPG, GRGDSP, and GGGGRGDSY.^[^ ^23^ ^]^ These peptides must be conjugated with biomaterials to effectively perform their biological functions.^[^ ^29^ ^]^ Synthetic RGD‐containing peptides have been widely used in cartilage tissue engineering to functionalize a wide range of biomaterials. These consist of sodium alginate, agarose hydrogel, microcavitary alginate hydrogel, biodegradable polyethylene glycol, and cellulose‐binding domain, which is derived from the cellulobiohydrolase I of the transgenic fungus Trichoderma koningi.^[^ ^23^ ^]^ For example, the incorporation of RGD in immobilized alginate scaffolds has been demonstrated to markedly enhance chondrogenesis, as evidenced by the elevated mRNA expression of *Sox9*, *Col2*, and *Acan* in comparison to that observed in alginate scaffolds.^[^ ^30^ ^]^ However, the ability of these peptides to enhance in vitro or in vivo chondrogenesis has primarily been demonstrated in the presence of the chondroinductive growth factor TGF‐β3. This suggests their chondroconductivity rather than chondroinductivity—a property that enables the independent induction of differentiation in, undifferentiated, primitive, and pluripotent cells into the cartilage‐forming cell lineage.^[^ ^23^ ^]^ A key criterion for chondroinductivity is the ability of bioactive agents or biomaterials to induce cartilage formation in vivo at ectopic sites, such as subcutaneous or intramuscular locations.^[^ ^23^ ^]^

Chondroinductive growth factors (e.g., BMP‐2 and TGF‐β3) and their signaling‐transduction components are another major resource to design and develop chondrogenic peptides. BMP‐2 has long been recognized as a potent inducer of bone formation through endochondral ossification. It may also directly activate intramembraneous ossification in certain delivery modes and mechanical stability.^[^ ^16b^ ^]^ Therefore, when being used as a chondroinductive growth factor, BMP‐2 may further stimulate chondrocyte hypertrophy and ossification, thereby compromising cartilage structure and functions.^[^ ^31^ ^]^ To approach this problem, casein kinase II (CK2), a cytoplasmic enzyme to phosphorylate BMP receptor type‐IA and activate downstream BMP signaling, was used to develop a novel chondrogenic peptide CK2.1.^[^ ^18^, ^32^ ^]^ In the micromass chondrogenesis in vitro model of C3H10T1/2 cells, CK2.1 exhibits a comparable efficacy to BMP‐2 in enhancing Smad binding element luciferase activity and Alcian blue staining.^[^ ^18^, ^33^ ^]^ When administered via tail vein injection and into knee cartilage defects in C57BL/6J mice, CK2.1 remarkably increased cartilage thickness and upregulated the expression of Col2 and Col9 in femoral articular cartilage.^[^ ^18^, ^33^ ^]^ Most importantly, unlike BMP‐2, CK2.1 did not induce Col10 expression or affect trabecular bone mineral density.^[^ ^18^, ^33^ ^]^ However, there is still a lack of robust evidence, such as the induction of ectopic cartilage formation, to prove its chondroinductivity. In contrast to BMP‐2, TGF‐β3 has long been regarded as a chondroinductive growth factor^[^ ^1^ ^]^ and is commonly used as an indispensable component in chondrogenic medium. A peptide with the sequence of SPPEPS has been developed from the latency‐associated protein region^[^ ^34^ ^]^ — a ligand for numerous integrins.^[^ ^35^ ^]^ Similar as the peptides derived from ECM or cell–cell adhesion molecule, this peptide is only chondroconductive. Therefore, hitherto, there has been no experimentally proven chondroinductive peptides.

In the present study, using an ectopic cartilage model of erector spinae SD rats, BMP‐2 induced de novo bone formation without detectable cartilage formation. While TGF‐β3 induced not only a tremendous amount of de novo cartilage formation but also de novo bone formation. Among all TPs, only TP8 induced significant cartilage formation with a zonal structure and a thick deep zone, which proved its chondroinductivity (Figure 1). Furthermore, no new bone formation was detected, which proved that TP8 was not osteoinductive (Figure S2, Supporting Information). We further showed that TP8 significantly enhanced higher secretion of chondrogenic differentiation markers (GAGs, PGs, Sox9, Col2, and Acan) in the absence of TGF‐β3, which further corroborated its chondroinductivity (Figures 2 and 4). In our study, 500 ng mL^−1^ TP8 bore a comparable capacity in inducing GAGs and PGs as 10 ng mL^−1^ TGF‐β3 at their optimal concentrations (Figure 2). To our best knowledge, TP8 is the first experimentally proven chondroinductive peptide.

Interestingly, 500 ng mL^−1^ TP8 induced significantly higher levels of *Sox9* gene expression and much lower levels of *Col10* gene in comparison with 10 ng mL^−1^ TGF‐β3 (Figure 4A). Sox9 is a master transcriptional factor for transactivating chondrogenesis and is essential for the transition from MSCs to early chondrocytes.^[^ ^36^ ^]^ Col10 is chiefly found in hypertrophic chondrocytes within cartilage, with its localization primarily in the calcified zone of articular cartilage in long bones and the hypertrophic zone of the growth plate.^[^ ^37^ ^]^ Furthermore, TGF‐β3 significantly increased the gene expression of *Col1a1* and *Col1a2* in comparison with the Control group, while TP8 didn't significantly change the expression of *Col1a1* gene and *Col1a2* gene. Col1, a fibrillar collagen, is the most sufficient protein in mammals, especially in bone tissue. 90% of the organic component of the bone matrix is made up of Col1. Col1 is synthesized, deposited, and remodeled by mesenchymal cell lineages during bone tissue development, formation, and homeostasis.^[^ ^38^ ^]^ Our results suggested that TP8 is prone to induce early rather than terminal chondrogenic differentiation or new bone formation.

In animal studies, we further investigated the efficacy of TP8 in repairing the cartilage defect of rabbit medial femoral condyles. The cartilage thickness in the Blank group was less than that of surrounding cartilage tissue, which indicated an insufficient cartilage regeneration. The presence of hydrogel (Control group) did not significantly enhance the cartilage thickness. In comparison, the cartilage thicknesses in the groups of both TGF‐β3 and TP8 were significantly higher than that of the Blank group, which indicated they could significantly enhance cartilage regeneration. Meanwhile, cartilage thickness in the TP8 group but not in the TGF‐β3 group was significantly higher than that of the Control group (Figure 10A,B). Additionally, ICRS macroscopic analysis, histological analysis, and immunohistochemical staining analysis confirmed that TP8 exhibited a comparable efficacy in repairing the cartilage defects as TGF‐β3 (Figures 8, 9, and 11). A typical articular cartilage constitutes a zonal structure with different zones, such as superficial zone, middle or transition zone, deep or radial zone, and calcified zone in order from the surface of articular cartilage to subchondral bone.^[^ ^1^, ^39^ ^]^ The superficial zone, which comprises high‐density flat chondrocytes, accounts for 10% to 20% of the articular cartilage thickness.^[^ ^40^ ^]^ The middle zone accounts for 40% to 60% of the total cartilage volume and is populated by spherical chondrocytes at low density.^[^ ^1^ ^]^ The deep zone accounts for between 30% and 40% of the total amount of articular cartilage and contains characteristic “chondron” — peri‐cellular matrix‐surrounded large chondrocytes with a columnar alignment.^[^ ^40^, ^41^ ^]^ Due to the perpendicular orientation of collagen fibrils to the articular surface, the deep zone offers the highest resistance against compressive pressures. The calcified zone is located between the cartilage and the subchondral bone and contains a sparse cell population with hypertrophic chondrocytes.^[^ ^42^ ^]^ In this study, histomorphometric analysis showed that TP8 induced a significantly thicker layer of deep zone and thinner calcified zone than TGF‐β3, which indicated that TP8 induced an early chondrogenic differentiation and chondrocytes maturation rather than terminal hypertrophic differentiation and calcification (Figure 10C–E).

To further illustrate the molecular mechanisms for the chondroinductive effect of TP8, we investigated the transmembrane receptors and major transcriptional factors. In the TGF‐β superfamily, there are many ligands, such as TGF‐βs, BMPs, growth differentiation factors, the müllerian inhibiting substance, activins, and nodal. Accordingly, there are three types of receptors (seven type I receptors, five type II receptors, and one type III receptors) to mediate their intracellular signaling. Type I receptors include activin receptor‐like kinase 1–7 (ALK 1–7), and type II receptors include TGFβR2, BMPR2, ACVR2, ACVR2B and AMHR2.^[^ ^19^ ^]^ TGF‐β3 especially binds and signals through ALK5 and TGFβR2 that couple to and activate Smad 2/3. In contrast, BMP‐2 binds and signals through ALKs (1, 2, and 3), BMPR2, ACVR2, and ACVR2B that couple to and activate Smad 1/5/8.^[^ ^43^ ^]^ In this study, we reconstructed the 3D structure of TP8 from its sequence and performed protein–protein molecular docking of TP8 with TGFβ receptors and obtained the Autodock vina docking score (Figure 5B–D). The docking scores of TP8 with those nine TGFβ receptors (ALK2, ACVR2, ALK4, ALK5, ACVR2B, ALK3, TGFβR2, BMPR2, and ALK1) were all lower than −5.0, suggesting that TP8 had certain binding activity with the above TGFβ receptors. The binding energy of ALK2 and ADVR2 with TP8 was the most negative, which suggested that TP8 might preferably bind with these two receptors. The different combinations of receptors may contribute to the different functional patterns of TP8 in comparison with TGF‐β3.

Very interestingly, western blot results showed that TP8 dramatically enhanced the level of p‐Smad1/5 by 2.5‐folds while there is no significant difference on the expression of p‐Smad2/3 (Figure 4D,E). Smad2/3 and Smad1/5/8 signaling play distinct but crucial functions in the biology of chondrocytes. ECM is regulated pro‐hypertrophic when TGF‐β3‐mediated Smad1/5/8 signaling is activated, whereas TGF‐β3‐mediated Smad2/3 signaling activation leads to anti‐hypertrophic and anti‐inflammatory effects.^[^ ^44^ ^]^ However, this is in conflict with our results that TP8 activated p‐Smad1/5/8, while didn't show significant pro‐hypertrophic effects. We hypothesized that other receptors and signaling pathways might also be involved in the chondroinductive effect of TP8. We further performed RNA sequencing and bioinformatic analysis to illustrate potential receptors. Our results demonstrated that TP8, similar to TGF‐β3, could upregulate genes involved in cartilage development, regulation, and connective tissue development (Figure 6C). Furthermore, 111 mRNAs were upregulated and 44 mRNAs were downregulated in the overlap of TGF‐β3‐ treated mouse BMSCs and TP8‐ treated mouse BMSCs compared with the untreated mouse BMSCs (Figure 6B). In this study, we compared chondroinductive efficacy of TP8 and TGF‐β3 at their respective optimal mass concentrations. Since the molecular weights of the two molecules are very different from each other, the comparisons presented in the study were made at different molecular concentrations. Cautions should be taken to extrapolate the present data to analyze the molecular mechanisms. Among the results, Lrp1 attracted our attention since it is a transmembrane endocytosis receptor and could be significantly updated by both of TP8 and TGF‐β3 (Figure 6D). Lrp1, a transmembrane endocytosis receptor, is expressed at a high level in articular cartilage chondrocytes and BMSCs and protects the matrix in joints.^[^ ^25^, ^45^ ^]^ Lrp1 primarily exerts its role in chondrogenic differentiation by modulating Axin2 promoter activity to activate the WNT/β‐catenin signaling pathway.^[^ ^46^ ^]^ In addition, Lrp1 could directly regulate WNT4 expression in chondrocytes through TGF‐β1 signaling.^[^ ^47^ ^]^ We showed that the knockdown of Lrp1 nullified the promoting effect of TP8 on the expression of proteins and genes related to chondrogenesis (Sox9) (Figure 6E–J). The molecular mechanisms of TP8 still need to be further investigated.

## 4 Conclusion

In this study, we successfully develop a novel peptide TP8 from TGF‐β3 and provide robust evidence to prove its chondroinductivity without osteoinductivity. 500 ng mL^−1^ TP8 showed a comparable capacity as 10 ng mL^−1^ TGF‐β3 in enhancing GAGs and PGs secretion in the micromass of BMSCs and significantly promoted the expression of chondrogenic marker (Sox9, Acan, and Col2) in comparison with the Control group. TP8 (500 ng mL^−1^) induced a significantly higher expression of the *Sox9* gene than 10 ng mL^−1^ TGF‐β3. Moreover, TP8 significantly upregulated the phosphorylation of Smad1/5 but not MAPK/JNK or Smad 2/3. The knockdown of Lrp1 nullified the TP8‐induced Sox9 expression. In rabbit medial femoral condyles with critical‐size cartilage defects, TP8 could induce neo‐cartilage formation with a significantly wider deep zone in comparison with the TGF‐β3 and Control. This is, to our best knowledge, the first study to develop and prove a novel chondroinductive peptide.

## 5 Experimental Section

### Peptide Design and Synthesis

The regions of high‐affinity binding between TGF‐β3 and its receptors were identified as α1, β2, β3, β4, β7, and β8 through analysis of the crystallographic data concerning the critical functional domains of TGF‐β3 and its type II receptors.^[^ ^24^, ^48^ ^]^ According to the protein structure orientation, ten TPs (**Table** **2**) were designed by exploring different permutations and combinations of functional regions and adjusting the length of the amino acid sequence between these regions. The peptides were manufactured by solid‐phase peptide synthesis with a purity of 95% in Huibo Co., Ltd (Hangzhou, China).

**Table 2:** Peptides Amino acid sequences of TPs (TP 0–9).

Peptides	Amino acid sequences of TPs
TP0	TILYYVGRTKIEQGPGGDYIDFRQDLGWKWVHEPKGYYA
TP1	TILYYVGRTGPGGDRQDLGWKWVHEPKGYY—————‐
TP2	—–YYVGRTGPGGDRQDLGWKWVHEPK———————‐
TP3	—LYYVGRT————RQDLGWKWVHEPK———————‐
TP4	—————–GPGGDRQDLGWKWVHEPKGYY—————‐
TP5	TILYYVGRTGPGGDRQDLGWK————————————
TP6	—————————‐RQDLGWKWVHEPKGYY—————–
TP7	TILYYVGRTGPGGD—————————————————
TP8	TILYYVGRT—————————WVHEPKGYY—————‐
TP9	TILYYVGRTGPGGD—————‐WVHEPKGYY—————‐

### Cell Culture

Mouse BMSCs were obtained from Cyagen Co., Ltd (Guangzhou, China). BMSCs were cultured in a proliferation medium consisting of Dulbecco's modified Eagle's medium (DMEM) (Gibco, Thermo Fisher Scientific, USA), 10% fetal bovine serum (FBS, Gibco), penicillin (100 units mL^−1^), and streptomycin (100 mg mL^−1^) (Gibco). The cells were kept at 37 °C in an environment with 100% relative humidity and 5% CO_2_. The medium was changed every 2 or 3 days. For experiments, cells of passages 3 to 6 were used. After reaching ≈80% confluency, cells were detached for further in vitro cell experiments.

### Cell Viability and Proliferation Assay

The viability of BMSCs was assessed by CCK‐8 assay (Dojindo Laboratories, Kumamoto, Japan). The BMSCs were seeded in 96‐well plates at a density of 6 × 10^3^ (6 K) cells well^−1^. At the selected time points (24 and 72 h), the culture medium was replaced with CCK‐8 solution (10 µL of CCK‐8 solution with 90 µL of culture medium). After incubation at 37 °C for 3 h, the optical density was measured at 450 nm wavelength in a microplate reader (ThermoFisher, USA). The proliferation of BMSCs was tested by Hoechst staining assay (Solarbio life science, Beijing, China). The BMSCs were seeded in 24‐well plates at a density of 50 × 10^3^ (50 K) cells well^−1^. At the selected time points (24 and 72 h), the culture medium was replaced with Hoechst solution (50 µL of Hoechst solution with 500 µL of culture medium). After incubation at 37 °C for 20 min, measurements were taken. The fluorescence microscope (Thermo Fisher Scientific, Shanghai, China) was used to visualize the cells at 350/460 nm wavelength. Images were analyzed using Image‐Pro Plus software.

### Chondrogenic Induction and Histological Evaluation of Micromasses

A 3D micromass culture system was utilized during the chondrogenic differentiation of BMSCs in vitro. In short, 500 × 10^3^ (500 K) BMSCs were collected as a cell suspension, put into a 15‐mL centrifuge tube, and centrifuged to generate a micromass. The chondrogenic medium (CM) was composed of MSC chondrogenic differentiation basal medium, sodium pyruvate, ascorbate‐2‐phosphate, *L*‐proline, dexamethasone, and ITS+ supplement, which were obtained from Cyagen Co., Ltd (Guangzhou, China). Micromasses were assigned to the following groups: the TGF‐β3 group (10 ng mL^−1^), the BMP‐2 group (500 ng mL^−1^), the TPs (500 ng mL^−1^) group, and TP8 (0, 10, 50, 100, and 500 ng mL^−1^), in which micromasses were incubated with the CM. The culture medium was changed every 3 to 4 days for a period of 14 days before undergoing histological processing.

Glycosaminoglycans (GAGs) and proteoglycans (PGs) formation in the extracellular matrix after BMSCs culture was determined by Alican blue staining and Safranin O staining on day 14. GAGs and PGs belong to non‐collagenous elements in ECM, playing crucial roles in providing viscosity and compressive resistance to the ECM. GAGs and PG secretion were indicators of chondrogenic differentiation. GAGs synthesis was confirmed qualitatively by using Alican blue staining and PG synthesis was confirmed qualitatively by using Safranin O staining.^[^ ^49^ ^]^ Briefly, micromasses were washed three times in phosphate‐buffered saline (PBS), fixed for 30 min in 4% paraformaldehyde, followed by paraffin embedding, sectioning, and staining with Alican blue (Cyagen Biosciences, Guangzhou, China) and Safranin O (TMS‐009, Sigma ‐Aldrich, USA) for 30 min at room temperature. The light microscope was used to conduct histological and histomorphometric investigations. Newly secreted GAGs and PGs were visualized under a light microscope. The histological results of micromasses were then scored according to the Visual Histological Grading System by three investigators (W.Z., Q.L., and S.J.)^[^ ^25^, ^50^ ^]^ (Table S3, Supporting Information). The area and integrated optical density of GAGs and PGs secretion were analyzed using Image‐Pro Plus software. The secretion levels of GAGs and PGs were calculated using the formula: mean optical density = IOD sum/area sum.

### qRT‐PCR Analysis

An RNA extraction kit (Takara Biotechnology, Japan) was used to isolate total RNA from BMSCs. PrimeScript RT reagent Kit (Takara Biotechnology, Japan) was used to reverse‐transcribe 1 µg of total RNA into complementary (c) DNA. Reverse‐transcribed cDNA was subjected to qPCR using SRY‐box transcription factor 9 (Sox9), collagen II (Col2), collagen X (Col10), collagen I (Col1), aggrecan (Acan), low‐density lipoprotein receptor (LDLR) ‐related protein‐1 (Lrp1) and glyceraldehyde 3‐phosphate dehydrogenase (GAPDH). **Table** **3** lists the primers used in qRT‐PCR. Values of each gene expression were normalized for GAPDH.

**Table 3:** Primers used for real‐time PCR.

Gene	Forward primer (5′‐3′)	Reverse primer (5′‐3′)
*Sox9*	GTGGACATCGGTGAACTG	GGTGGCAAGTATTGGTCAA
*Aggrecan*	ATGGCAACATTCACCTCTG	TAGCACTACCTCCGACATAG
*Col2*	GTCAATAATGGGAAGGCGGGAGG	CGAGGGCAACAGCAGGTTCACAT
*Col10*	GAATGCCTGTGTCTGCTT	TCATAATGCTGTTGCCTGTT
*Col1a1*	CGATGGATTCCCGTTCGAGT	TTCGATGACTGTCTTGCCCC
*Col1a2*	AATGGTGGCAGCCAGTTTGA	TCCAGGTACGCAATGCTGTT
*Lrp1*	GCGGTGTGACAACGACAATG	AGCCACCAGGAGGTCTTGTA
*GAPDH*	TGTGTCCGTCGTGGATCTG	TTGCTGTTGAAGTCGCAGGA

### Western Blot Analysis

Total protein from the BMSCs culture was extracted by using a radioimmunoprecipitation assay (RIPA) lysis buffer and 10% sodium dodecyl sulfate‐polyacrylamide gel electrophoresis was used to isolate the 20 µg of protein. Subsequent transfer to a polyvinylidene fluoride (PVDF) membrane (Millipore, Billerica, MA, USA), QuickBlock Blocking Buffer (Beyotime Biotechnology, China) was used to block the membrane and then incubated with primary antibodies against SOX9 (Abcam), COLLAGEN II (Abcam), AGGRECAN (ACAN) (Abcam), SMAD2/3 (Abcam), p‐SMAD2/3 (Abcam), SMAD1 (Abcam), SMAD5 (Abcam), p‐SMAD1/5 (Abcam), MAPK/JNK (Cell Signaling Technology), p‐MAPK/JNK (Cell Signaling Technology), LRP1 (Cell Signaling Technology), GAPDH (Abcam), and β‐ACTIN (Cell Signaling Technology) in 1: 1000 dilution overnight at 4 °C. The corresponding secondary antibodies (Cell Signaling Technology) were incubated with in 1:3000 dilution for 1 h. ImageJ software was used to quantify the final results of Western blot analysis. Values of each protein expression were normalized with GAPDH or β‐actin.

### Fluorescence Analysis of TP8 Localization in BMSCs

Fluorescently‐labeled (Rhodamine) TP8 (B‐TILYYVGRTWVHEPKGYY) with a purity of 98% was manufactured by solid‐phase peptide synthesis in SynPeptide Co., Ltd (Nanjing, China). The BMSCs (20 × 10^3^ (20 K) cells/coverslip) were seeded on glass coverslips (8 mm diameter) plated in a 48‐well plate and treated with 0 and 500 µg mL^−1^ of fluorescently‐labeled (Rhodamine) TP8 for 2 h. BMSCs were washed three times with PBS and fixed with 4% fresh paraformaldehyde for 10 min at room temperature. The cells were permeabilized using Triton X‐100 (0.5% in PBS) for a period of 5 min. To prevent non‐specific binding, cells were incubated with bovine serum albumin (BSA) (1% in PBS) for 30 min at room temperature. The cells were then stained for 1 h at room temperature in the dark using FITC‐Phalloidin. Cells were washed and subsequently counterstained for 5 min using Hoechst solution. Images were then captured using a Leica TCS SP8 confocal laser scanning microscope (CLSM) with a 63× objective lens (Leica, Germany).

### Molecular Docking

Protein–protein docking was used for molecular docking simulations of TP8 with TGF‐β receptors (ALK2, ACVR2, ALK4, ALK5, ACVR2B, ALK3, TGFβR2, BMPR2, and ALK1). Utilizing the PYMOL database to acquire 3D structure files for TP8 and using the Uniprot database to download 3D structure files for TGF‐β receptors, and then PYMOL was used to visualize them. For docking analysis, Mgtools 1.5.6 was used to convert the TP8 and TGF‐β receptor files into PDBQT format, excluding all water molecules and adding polar hydrogen atoms. The grid box was centered to encompass the domain of each protein and allow for free molecular movement. Autodock Vina 1.2.2 was used for molecular docking tests. The binding energy was utilized to estimate the binding affinity of TP8 and TGF‐β receptors. A more negative Autodock Vina docking score indicated a higher affinity binding of the peptide with the protein.^[^ ^51^ ^]^

### High‐Throughput Generation Sequencing

Total RNA was isolated from BMSCs at 24 h using TRIzol reagent (Invitrogen, USA). The purity of RNA was checked using a NanoPhotometer spectrophotometer (Thermo Fisher Scientific, USA), and the concentration of RNA was determined using the Qubit RNA assay kit on the Qubit 2.0 Fluorometer (Life Technologies). Using the RNA Nano 6000 assay kit from the Bioanalyzer 2100 System (Agilent Technologies, USA), the integrity of RNA was evaluated. Using standard Illumina Truseq kits (NEB, USA), polyadenylated RNA‐seq was carried out, and the Illumina HiSeq 4000 PE150 platform was used to analyze the sequencing data that were obtained.

The “fastq” file of RNA‐seq data was aligned by using STAR. RNA expression was calculated by using RSEM and normalized by using TPM. Transcript per million (TPM) values and the count of reads mapped to each gene were determined using RNA‐Seq by Expectation‐Maximization (RSEM). Differential expression analysis between the two groups was performed using the “edgeR” R software package. With the cut‐off of *P*‐value < 0.05 and FDR < 0.25 and |log2(fold change)| > 1, the RNAs were considered as significantly expressed RNA. “pheatmap” and “ggplot2” R package were used for data visualization. “GO” and “KEGG” were used for pathway enrichment analysis.

### siRNA‐Mediated Knockdown of Lrp1

RNA interference was used to knock down the Lrp1 gene and protein production. The lentiviral Lrp1 siRNAs were purchased from GenePharma (Shanghai, China). Table 1 lists the siRNA sequences. Prior to transfection, the cells were transferred in 12‐well plates at a density of 70 × 10^3^ (70 K) cells well^−1^. The transient transfections with a 20 µm concentration of Lrp1 siRNAs and a 20 µm nonspecific oligoduplex (NC, nonsilencing control) were performed by using GP‐transfect‐Mate Transfection Agent (GenePharma, Shanghai, China). At 6 h after the transfection, the medium was exchanged for fresh medium, and the cells were cultured for another 1, 3, 4, and 7 days. The mRNA and protein of the cells were extracted after 1, 3, 4, and 7 days. The second transfection was performed 4 days after the first transfection.

### Hydrogel Preparation and In Vitro Culture of TP8‐Functionalized Gel‐MA (Gelatin‐methacryloyl) Hydrogels

First, gelatin with methacrylic anhydride (Gel‐MA) was synthesized as described in the previous report, where detailed modification procedures are provided.^[^ ^52^ ^]^ Second, 20% (w/v) precursor solutions were obtained by dissolving the freeze‐dried Gel‐MA macromer in PBS as it was produced. Gel‐MA and 0.5% (w/v) photoinitiator 2‐hydroxy‐1‐(4‐(hydroxyethoxy) phenyl)‐2‐methyl‐1‐propanone (Irgacure 2959, Sigma ‐Aldrich, USA) (50 mg ml^−1^ in 75% ethanol solution) were mixed in predetermined proportions, vortexed for 30 s, and then exposed to full UV light (HTLD‐4II, Shenzhen Height‐LED Opto‐electronic Tech, Ltd, China). The TP8‐functionalized Gel‐MA hydrogels were prepared by adding TP8 (500 ng mL^−1^) to the precursor solution and the Control group means Gel‐MA hydrogels without TP8. The calcein AM (Live) and ethidium bromide (Dead) staining were used to analyze the viability of BMSCs‐treated with TP8‐functionalized Gel‐MA hydrogels.

### Validation of Beneficial Effects of TP8 In Vivo

All animal experiments were approved by the Ethical Review Committee of Animal Experiments in Guangdong Medical Devices Quality Surveillance and Test Institute (acceptance number: JL033‐03, date: 2020.10.20) and Guangdong Huawei Testing Co., LTD (acceptance number: 20 211 002, date: 2021.01.04).

Rat ectopic osteogenesis and cartilage model construction: Haiao oral repair sponge (ZH‐bio, China) was used as a scaffold material. The Haiao oral repair sponge was cut into discs with a 5 mm diameter puncher that was sterilized by autoclaving. The sponge was filled with a sterile solution of TP 0–9, BMP‐2, and TGF‐β3 (10 µL of 5 µg µL^−1^ each), and the sterile culture hood was used to let the discs to dry. The male Sprague–Dawley (SD) rats (180–220 g) were anesthetized and 1.5–2.0 cm sagittal incisions were made on thier backs. Vertical muscles were separated bluntly to create defects, into which the grafts were implanted (*n* = 4 per group). Post‐surgery, antibiotics were administered via intramuscular injection to the above rats (Figure S4, Supporting Information).

Rabbit cartilage defect model construction: GelMA hydrogel was used as a scaffold material. Adult male New Zealand white rabbits (2–2.5 kg, 3–4 months old), were selected randomly for the experiment, were fed in a clean animal room for 1 week so that they could adapt to the environment, and then the hair on the joints of rabbits was removed, followed by injection with 3% (w/v) pentobarbital (1 mL kg^−1^) through the ear vein. Full‐thickness cartilage defects with diameters of 4 mm and a depth of 2 mm. Subsequently, the hydrogels were implanted in the defect. UV radiation (365 nm, 90 s) photopolymerized the Gel‐MA prepolymer solution. Finally, the patella was reset, and the wound was sutured layer by layer (Figure S5, Supporting Information). Following the surgical operation, the rabbits were administered antibiotics via intramuscular injection. Four groups were set up: the Blank group (no implant materials), the Control group (treated with Gel‐MA hydrogels without TGF‐β3 or TP8), the TGF‐β3 group (treated with TGF‐β3 – functionalized Gel‐MA hydrogels), and TP8 group (treated with TP8 – functionalized Gel‐MA hydrogels).

### Cartilage Regeneration and Cartilage Defect Healing Analysis

Four weeks post‐implantation, all rats and rabbits were euthanized for analysis. Micro‐CT system (SkyScan1172, Bruker, Belgium) was used to scan the collected specimens and perform the radiographic analysis. The scanned volumes had a voxel resolution of 10 µm, with each volume consisting of 500 slices. The images obtained were utilized to reconstruct tomograms using the 3D Creator software. After fixing in 4% paraformaldehyde for 1 week and dehydrated step by step, the specimens were embedded and sliced to 4 µm thickness, stained with Masson trichrome, hematoxylin and eosin (H&E), Safranin O, and Toluidine blue. For immunohistochemical staining, sections were deparaffinized and blocked in 3% hydrogen peroxide solution for 10 min. Sections were then incubated overnight at 4 °C with primary antibodies, including COLLAGEN II (Cohesion Biosciences,) and AGGRECAN (ACAN) (Cohesion Biosciences). Correspondingly, sections were followed by secondary goat anti‐mouse antibody (Nanjing KeyGen Biotech. Co., Ltd., China) at 55 °C for 30 min and detected with 3,3′ ‐diaminobenzidine tetrahydrochloride (DAB) solution for 10 min. Thereafter, the sections were stained with Mayer's hematoxylin as a counterstain (Sigma‐Aldrich) for 8 min and mounted with neutral balsam. The light microscope was used to visualize the formation of new cartilage and the repair of cartilage defects. The percentage of positive area and IOD of Acan and Col2 expression were evaluated using Image‐Pro Plus software. Acan and Col2 expressions were calculated according to the following formula: mean optical density = IOD sum/area sum. Image‐Pro Plus software was used to analyze images from the three parts in each group.

Three investigators (W.Z., Q.L., and S.J.) independently scored the histological sections using two evaluation systems: ICRS macroscopic evaluation of cartilage repair^[^ ^25a,b^ ^]^ (Table S1, Supporting Information) and histological scoring system for evaluation of the repair of full‐thickness articular cartilage defect in rabbit^[^ ^25c^ ^]^ (Table S2, Supporting Information). The thickness of neo‐cartilage, deep zone, and calcified zone were measured using Image‐Pro Plus software. The average thickness of neo‐cartilage was calculated according to the following formula: Average thickness of neo‐cartilage = average thickness of neo‐cartilage / average thickness of surrounding normal cartilage. The average thickness of the deep zone or calcified zone was calculated according to the following formula: Average thickness of the deep zone or calcified zone/Average thickness of neo‐cartilage. Images from three sections per group and three different fields per section were analyzed using Image‐Pro Plus software.

### Statistical Analysis

All the in vitro experiments were performed at least 3 times. GraphPad Prism software version 9.5.1 was used to analyze the data and detect significant differences. All data were presented as mean value ± standard deviations (SD). Differences between the two groups were calculated using an unpaired two‐tailed Student's *t*‐test. Comparisons among three groups or more were made using one‐way ANOVA. Post‐hoc comparisons were made using Tukey's multiple comparisons. In all cases, *p* < 0.05 was considered to indicate a statistically significant difference, and *p* < 0.01, 0.001, and *p *< 0.0001 were considered highly significant differences.

### Ethics Approval and Consent to Participate

All animal experiments were approved by the Ethical Review Committee of Animal Experiments in Guangdong Medical Devices Quality Surveillance and Test Institute (acceptance number: JL033‐03, date: 2020.10.20) and Guangdong Huawei Testing Co., LTD (acceptance number: 20211002, date: 2021.01.04).

## Conflict of Interest

The authors declare no conflict of interest.

## Supporting information



Supporting Information

## Data Availability

The data that support the findings of this study are available from the corresponding author upon reasonable request.
