# Supplementary material for: TP8, A Novel Chondroinductive Peptide, Significantly Promoted Neo‐Cartilage Repair without Activating Bone Formation
Source: Adv Healthc Mater. 2024 Dec 17;14(6):2401752. doi: 10.1002/adhm.202401752 (PMC11874676; doi:10.1002/adhm.202401752)
Supplement: Supplementary file 1 — Supporting Information [file ADHM-14-0-s001.docx]

**Figure supplement**


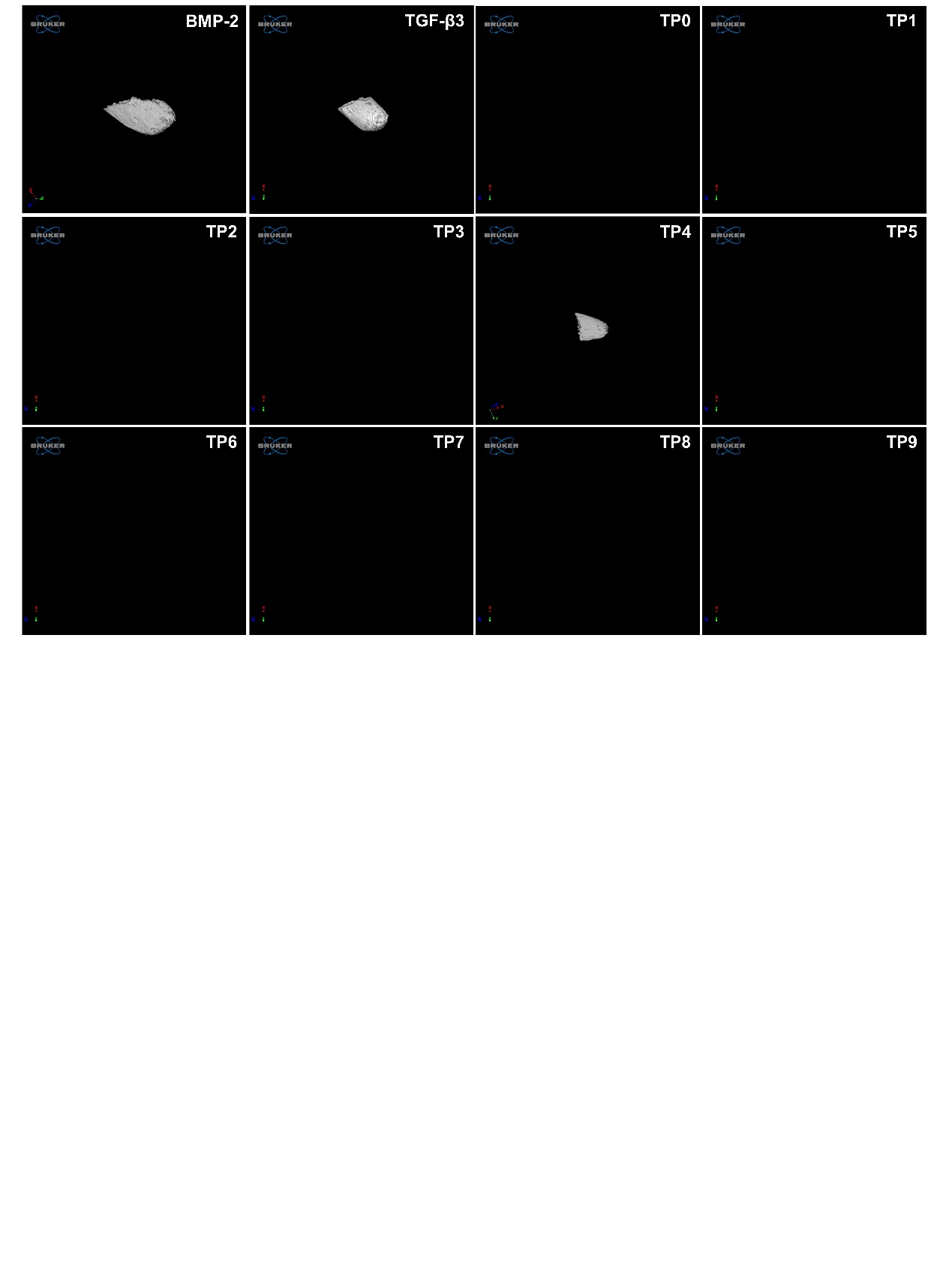


**Figure S1.** Micro CT images showing mineralized tissue formation in the groups of TGF-β3, BMP-2, and TP4 in the ectopic cartilage model of erector spinae male SD rats after 4 weeks post implantation. In contrast, no detectable mineralized tissue was detected in the other TP groups.


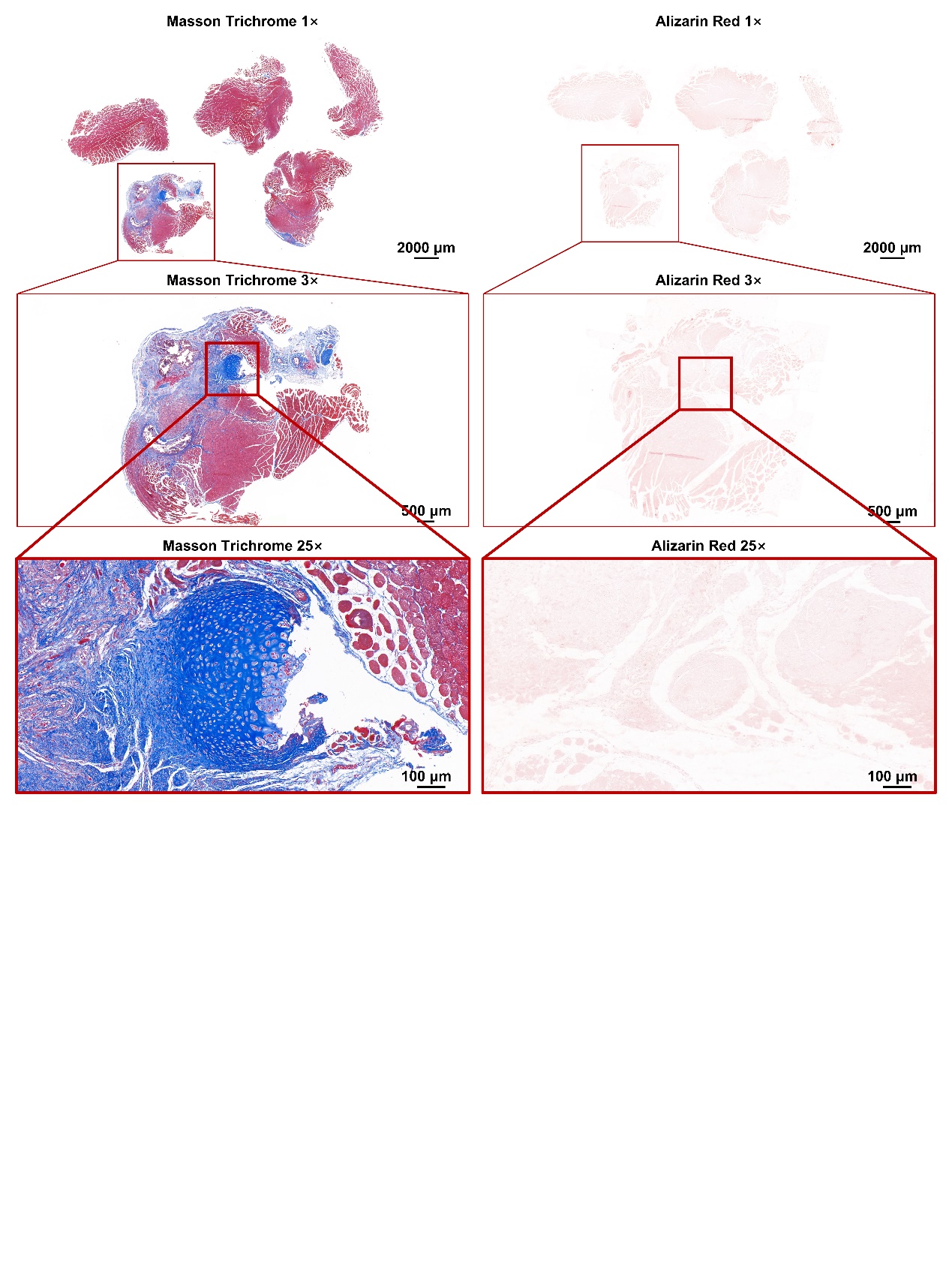


**Figure S2.** Rat ectopic osteogenesis and cartilage model construction: Haiao® oral repair sponge (ZH-bio, China) was used as a scaffold material. Cut the Haiao® oral repair sponge into discs with a 5 mm diameter puncher that has been sterilized by autoclaving. The sponge was filled with sterile solution of TP 8 (10 μL of 5 μg μL^−1^ each), and the sterile culture hood was used to let the discs to dry. The male Sprague-Dawley (SD) rats (180-220 g) were anaesthetized and 1.5-2.0 cm sagittal incisions were made on their backs. Vertical muscles were separated bluntly to create defects, into which the grafts were implanted (n = 4 per group). Post-surgery, antibiotics were administered via intramuscular injection to the above rats. 4 weeks post-implantation, all rats were euthanized for analysis. After fixing in 4% paraformaldehyde for 1 week and dehydrated step by step, the specimens were embedded and sliced to 4 μm thickness, stained with Masson trichrome and Alizarin Red. The light microscope was used to visualize the results. Light micrographs of Alizarin Red (×1, ×3 and ×25) and Masson Trichrome (×1, ×3 and ×25) stained cross-sections of the TP8 -containing collagen membrane that was implanted in erector spinae of Sprague-Dawley (SD) rats for 21 days. Scale bar in ×1= 2000 μm; scale bar in ×3= 500 μm; scale bar in ×25= 100 μm.


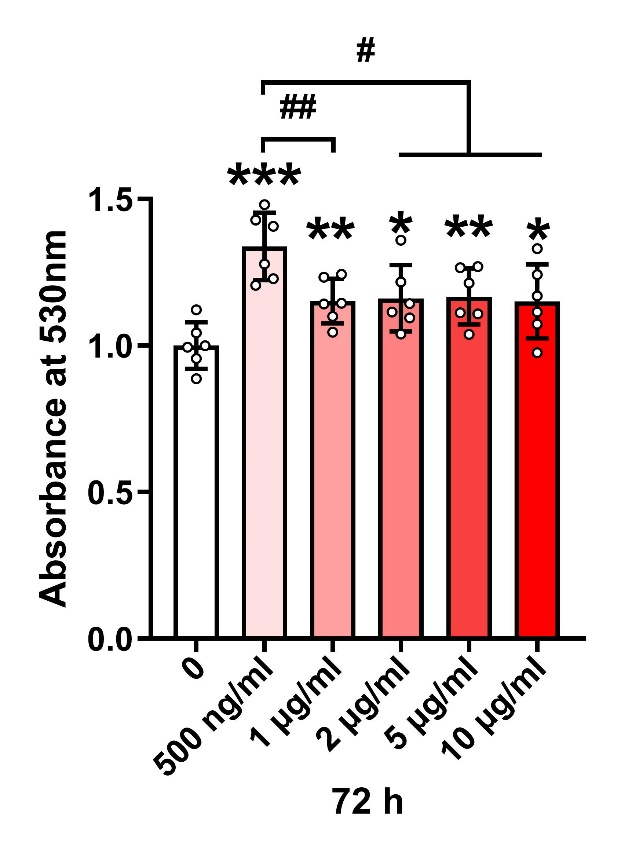


**Figure S3.** The cell proliferation of BMSCs was assessed using PrestoBlue^TM^ HS Cell Viability Reagent (ThermoFisher Scientific, USA). Mouse BMSCs were obtained from Cyagen Co., Ltd (Clinisciences, the Netherlands) and were cultured in a proliferation medium consisting of Dulbecco’s modified Eagle’s medium (DMEM) (Gibco, UK), 10 % fetal bovine serum (FBS, Gibco, UK), penicillin (300 μg mL^−1^) (Sigma-Aldrich, USA), streptomycin (250 μg mL^−1^) (Sigma-Aldrich, USA), and fungizone (1.25 μg mL^−1^) (Gibco, UK). The cells were kept at 37 °C in an environment with 100% relative humidity and 5% CO_2_. Cells of passage 4 were used for cell proliferation assay. The BMSCs were seeded in 96-well plates at a density of 6×10^3^ (6K) cells/well (n=9/group). The cells were treated without or with TP8 at 500 ng mL^−1^, 1 μg mL^−1^, 2 μg mL^−1^, 5 μg mL^−1^, or 10 μg mL^−1^. 72 h post-treatment, the culture medium was replaced with PrestoBlue solution (10 μL of PrestoBlue solution with 90 μL of culture medium). After an incubation at 37 °C for 1 h, the optical density was measured at 530 nm wavelength in a Synergy HT^®^ spectrophotometer (Bio Tek Instruments, USA). GraphPad Prism software version 9.5.1 was used to analyze the data and detect significant differences. The results showed that in comparison with the Control group (no TP8), TP8 at 500 ng mL^−1^, 1 μg mL^−1^, 2 μg mL^−1^, 5 μg mL^−1^, and 10 μg mL^−1^ significantly promoted cell proliferation. Interestingly, the effect of TP8 at 500 ng mL^−1^ was significantly higher than that at higher dosages (1 μg mL^−1^, 2 μg mL^−1^, 5 μg mL^−1^, or 10 μg mL^−1^). (Data were presented as mean value ± standard deviation (SD); statistical signiﬁcance was calculated using one-way ANOVA with a Tukey post-test; significant effect of the treatment: *p < 0.05 vs Control group, **p < 0.01 vs Control group, ***p < 0.001 vs Control group, ^#^p <0.05 vs TGF-β3, ^##^p <0.01 vs TGF-β3).


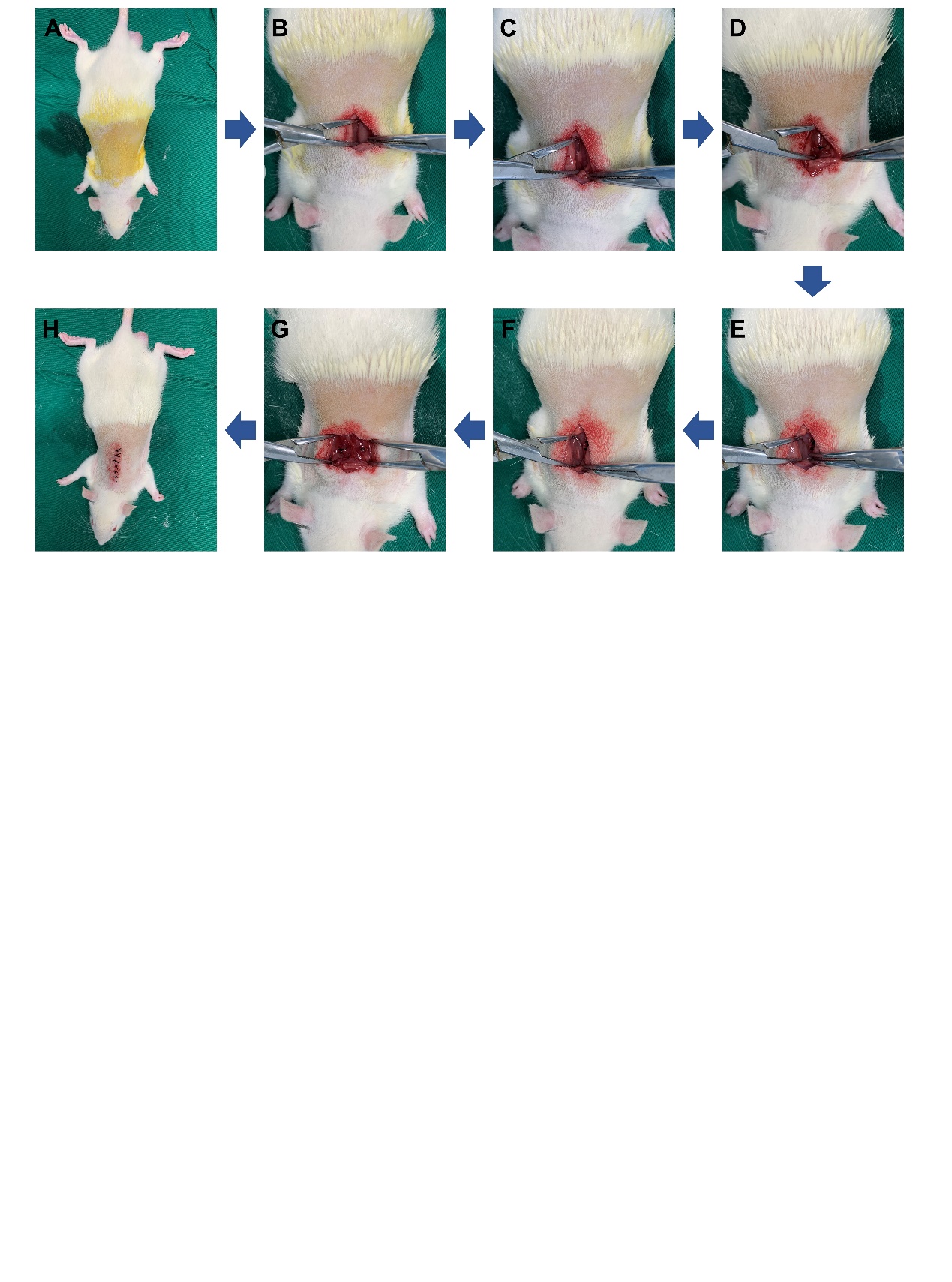


**Figure S4.** Rat ectopic osteogenesis and cartilage induction model construction. A) Anesthesia and shave the hairs on the back of SD rat. B) Pouch creation in the dorsal region on the one side. C) Implantation of the disc into a muscle bed and (D) suturing of the muscle pouch after a disc implantation. E) Pouch creation in the dorsal region on the other side. F) Implantation of the other disc into a muscle bed and (G) suturing of the muscle pouch after a disc implantation. H) Suturing the soft tissue wounds.


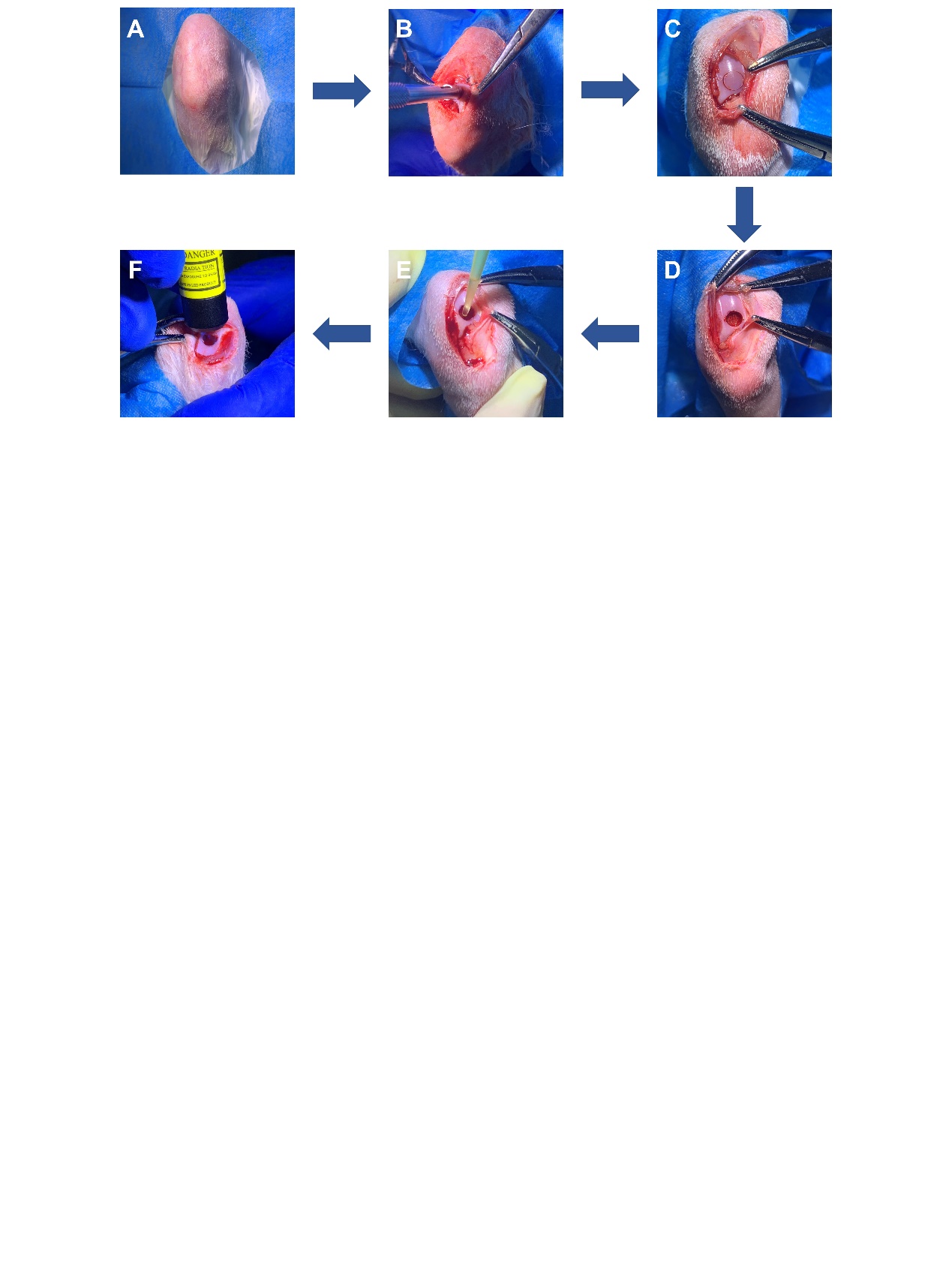
**Figure S5.** Rabbit cartilage defect model construction. A) Anesthesia and shave the hairs on the joints of rabbit. B) A medial para-patellar incision was made to dislocate the patella and expose the femur condyle. C, D) Full-thickness cartilage defects were created on the center of the trochlear groove using a drill. E) Implantation of hydrogels. F) The Gel-MA prepolymer solution was photopolymerized by UV light.

**Table supplements**

Table S1 ICRS macroscopic evaluation of cartilage repair^[25a, 25b]^

| **Categories** | **Scores** |
| --- | --- |
| Degree of defect repair |  |
| In level with surrounding cartilage | 4 |
| 75% repair of defect depth | 3 |
| 50% repair of defect depth | 2 |
| 25% repair of defect depth | 1 |
| No repair of defect depth | 0 |
| Integration to border zone |  |
| Complete integration with surrounding cartilage | 4 |
| Demarcating border < 1mm | 3 |
| 3/4th of graft integrated, 1/4th with a notable border > 1mm width | 2 |
| 1/2 of graft integrated with surrounding cartilage, 1/2th with a notable border > 1mm | 1 |
| From no contact to 1/4th of graft integrated with surrounding cartilage | 0 |
| Macroscopic appearance |  |
| Intact smooth surface | 4 |
| Fibrillated surface | 3 |
| Small, scattered fissures orcracs | 2 |
| Several, small or few but large fissures | 1 |
| Total degeneration of grafted area | 0 |
| Overall repair assessment |  |
| Grade I: normal | 12 |
| Grade II: nearly normal | 11-8 |
| Grade III: abnormal | 7-4 |
| Grade IV: severely abnormal | 3-1 |

Table S2 Histological scoring system for evaluation of the repair of full-thickness articular cartilage defect in rabbit^[25c]^

| **Histological scoring system** | **Scores** |
| --- | --- |
| (a) Overall defect evaluation (throughout the entire defect depth) |  |
| 1. Percent filling with newly formed tissue |  |
| 100% | 3 |
| >50% | 2 |
| <50% | 1 |
| 0% | 0 |
| (b) Subchondral bone evaluation (within the bottom 2 mm of defect) |  |
| 2. Percent filling with newly formed tissue |  |
| 100% | 3 |
| >50% | 2 |
| <50% | 1 |
| 0% | 0 |
| 3. Subchondral bone morphology |  |
| Normal, trabecular bone | 4 |
| Trabecular bone, with some compact bone | 3 |
| Compact bone | 2 |
| Compact bone and fibrous tissue | 1 |
| Only fibrous tissue or no tissue | 0 |
| 4. Extent of new tissue bonding with adjacent bone |  |
| Complete on both edges | 3 |
| Complete on one edge | 2 |
| Partial on both edges | 1 |
| Without continuity on either edge | 0 |
| (c) Cartilage evaluation (within the surface 1 mm of defect) |  |
| 5. Morphology of newly formed surface tissue |  |
| Exclusively articular cartilage | 4 |
| Mainly hyaline cartilage | 3 |
| Fibrocartilage (spherical morphology observed with >75% of cells) | 2 |
| Only fibrous tissue (spherical morphology observed with <75% of cells) | 1 |
| No tissue | 0 |
| 6. Thickness of newly formed cartilage |  |
| Similar to the surrounding cartilage | 3 |
| Greater than the surrounding cartilage | 2 |
| Less than the surrounding cartilage | 1 |
| No cartilage | 0 |
| 7. Joint surface regularity |  |
| Smooth, intact surface | 3 |
| Surface fissures (>25% of new surface thickness) | 2 |
| Deep fissures (<25% of new surface thickness) | 1 |
| Complete disruption of the new surface | 0 |
| 8. Chondrocyte clustering |  |
| None at all | 3 |
| <25% chondrocytes | 2 |
| 25-100% chondrocytes | 1 |
| No chondrocytes present (no cartilage) | 0 |
| 9. Chondrocytes and GAG content of new cartilage |  |
| Normal cellularity with normal Safranin O staining | 3 |
| Normal cellularity with moderate Safranin O staining | 2 |
| Clearly less cells with poor Safranin O staining | 1 |
| Few cells with no or little Safranin O staining or no cartilage | 0 |
| 10. Chondrocytes and GAG content of adjacent cartilage |  |
| Normal cellularity with normal Safranin O staining | 3 |
| Normal cellularity with moderate Safranin O staining | 2 |
| Clearly less cells with poor Safranin O staining | 1 |
| Few cells with no or little Safranin O staining or no cartilage | 0 |

Table S3 Visual histological grading system for micromass culture^[25c, 50]^

| **Visual histological grading system for micromass culture** | | |
| --- | --- | --- |
| A. Uniformity and darkness of stain | No stain | 0 |
|  | Weak staining of poorly formed matrix | 1 |
|  | Moderately even staining | 2 |
|  | Even dark stain | 3 |
| B. Distance between cells/amount of matrix accumulated | No spacing between cells | 0 |
|  | Cells<1 cell-size apart | 1 |
|  | Cells approx. 1 cell-size apart | 2 |
|  | Low cell density, >1 cell and an extensive matrix | 3 |
| C. Cell morphologies represented | Condensed/necrotic/pycnotic bodies | 0 |
|  | Spindle/fibrous | 1 |
|  | Mixed spindle/fibrous/rounded chondrogenic morphology | 2 |
|  | Majority rounded/chondrogenic | 3 |
